# Lung microbiome: new insights into the pathogenesis of respiratory diseases

**DOI:** 10.1038/s41392-023-01722-y

**Published:** 2024-01-17

**Authors:** Ruomeng Li, Jing Li, Xikun Zhou

**Affiliations:** 1 Department of Biotherapy, Cancer Center and State Key Laboratory of Biotherapy, West China Hospital, Sichuan University, Chengdu, 610041 China; 2 State Key Laboratory of Oral Diseases, National Clinical Research Center for Oral Diseases, Chinese Academy of Medical Sciences Research Unit of Oral Carcinogenesis and Management, West China Hospital of Stomatology, Sichuan University, Chengdu, Sichuan 610041 China

**Keywords:** Microbiology, Respiratory tract diseases, Inflammation, Lung cancer, Prognostic markers

## Abstract

The lungs were long thought to be sterile until technical advances uncovered the presence of the lung microbial community. The microbiome of healthy lungs is mainly derived from the upper respiratory tract (URT) microbiome but also has its own characteristic flora. The selection mechanisms in the lung, including clearance by coughing, pulmonary macrophages, the oscillation of respiratory cilia, and bacterial inhibition by alveolar surfactant, keep the microbiome transient and mobile, which is different from the microbiome in other organs. The pulmonary bacteriome has been intensively studied recently, but relatively little research has focused on the mycobiome and virome. This up-to-date review retrospectively summarizes the lung microbiome’s history, composition, and function. We focus on the interaction of the lung microbiome with the oropharynx and gut microbiome and emphasize the role it plays in the innate and adaptive immune responses. More importantly, we focus on multiple respiratory diseases, including asthma, chronic obstructive pulmonary disease (COPD), fibrosis, bronchiectasis, and pneumonia. The impact of the lung microbiome on coronavirus disease 2019 (COVID-19) and lung cancer has also been comprehensively studied. Furthermore, by summarizing the therapeutic potential of the lung microbiome in lung diseases and examining the shortcomings of the field, we propose an outlook of the direction of lung microbiome research.

## Introduction

With the Human Microbiome Project, the human body’s microbiome has gradually been unveiled.^1^ The microbiome includes all the microbes and their gene sequences (including homologous sequences) in a specific habitat at a specific time.^2,3^ It contains every organism, including not only bacteria but also archaea, fungi, and viruses. Various methods of obtaining DNA (metagenomics), RNA, metabolites, and proteins have been reported.^4–6^ In the past, the oropharyngeal microbiome and the gut microbiome have been studied with great enthusiasm, but the sterile environment of the lungs has been inherently perceived. The lung microbiome has thus gone unnoticed by the world. However, with the application of detection technologies, such as polymerase chain reaction (PCR), next-generation sequencing (NGS), and the maturation of DNA sequencing,^7,8^ researchers began to pay attention to the lung microbiome. The lung microbiome is mostly composed of bacteria, fungi, and viruses.^9,10^ The relationship between the lung microbiome and the oropharyngeal and gut microbiomes, especially the gut-lung axis, has been intensively studied. The gut-lung axis is bidirectional and influences the progression of intestinal and lung diseases in terms of metabolism, immunity, and other aspects. Although the exact mechanism is unknown, the lung microbiome has an impact on lung development. Germ-free rodents tend to have reduced lung parenchyma and less developed alveoli.^11^ The dominant population and abundance of the microbiome differ in healthy and diseased lungs.

Even the composition and size of the lung microbiome change dynamically under the influences of different kinds of diseases. For instance, in patients with asthma and COPD, pathogenic Proteobacteria, especially *Haemophilus*, were increased, whereas in cystic fibrosis (CF) patients, *Candida albicans* was increased.^12–14^ It follows that dysbiosis in the pulmonary microbiome, which imbalances the composition and size of the lung microbiome, affects disease occurrence, progression, and prognosis.^15^ Therefore, the lung microbiome can be considered an indicator of disease and diagnosis.

In addition, due to their geographical location, the lung microbiome is strongly related to the oropharyngeal and gut microbiomes. Numerous cases have confirmed the interaction between oral, gut, and lung microbes.^16–18^ If oral microorganisms enter the lungs and spread, they can directly form the lung microbial community and directly affect the growth of lung bacteria. However, this may lead to contamination of bronchoalveolar lavage fluid (BALF), causing limitations in the experimental results.^19–21^ To address this issue, sampling of the lung microbiome requires strict negative control methods^16,22^ and protective bronchial sampling techniques such as wax-sealed catheters.^23^

Since 2020, COVID-19 has broken out around the world, causing great damage to people’s health. Due to the constant changes in the coronavirus, it is clinically impossible to contain the infection and spread of the virus from the source. Researchers have provided insights into the link between the lung microbiome and this pandemic, and the results have been encouraging. By 2022, research on the lung microbiome will also gradually become linked to cancer. Changes in the lung microbiome are related to lung cancer occurrence, development, and prognosis. The lung microbiome composition differs between states, such that *Streptococci* and *Staphylococci* are more abundant in cancer patients, while the opposite is true in noncancerous subjects.^24^ Pulmonary microorganisms and their products affect clinical treatments, especially immunotherapy. Both immunotherapy and prognostic outcomes decreased with altered microbial abundance. Combining microbial therapy with lung cancer treatment may lead to an improvement in efficacy.

With technological progress, mysteries about the lung microbiome are gradually being unveiled. Studying microbial communities will further clarify the pathogenesis and development of many diseases. We will examine the history of the lung microbiome, the effects of the interactions between microbes and host factors, and explore new research directions.

## History of the lung microbiome

**Fig. 1:**
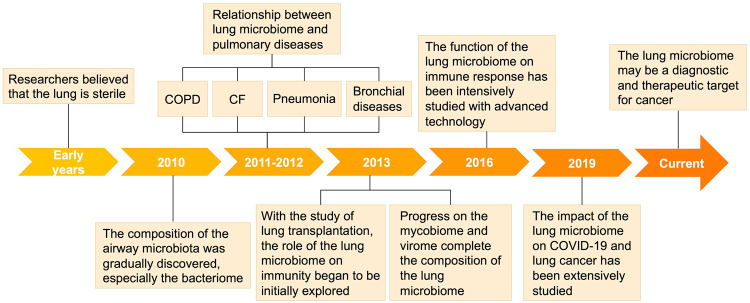
History of the lung microbiome. With the advancement of technology, studies on the lung microbiome were progressively funded and completed. In early years, the lung was believed to be a sterile environment; thus, its functions were ignored. In 2010, the composition of the lung microbiome, especially the bacteriome, was discovered, showing that the lung has its own microbiota. The relationship between the lung microbiome and pulmonary diseases has been uncovered. For example, COPD, CF, pneumonia, and bronchial disease were found to be closely associated with the lung microbiome in 2011 and 2012. With the study of lung transplantation, immunity, and the lung microbiome were shown to have a strong relationship. At the same time, scientists have paid attention to the mycobiome and virome, completing the composition of the lung microbiome. In 2016, the function of the lung microbiome in the immune response, especially the innate immune response, was intensively studied. From the end of 2019 to the present, researchers have found important connections to COVID-19 and lung cancer. The lung microbiome also plays an active role in these processes, and more studies are needed. (Figures are created with Servier Medical Art and exported under a paid subscription.)

The study of the lung microbiome is still in its infancy compared to that of the microbiomes of other parts of the body. In early years, scientists began investigating the effect of colonization on pulmonary allergy symptoms.^25^ However, most of the subsequent studies focused on the functions of the gut microbiome, fecal microbiome, etc., in the lung.^26^ It was evident that the lungs were in a sterile state, which was the perception of most people at that time. In 2010, researchers successively determined the composition of the airway microbiota; thus, the microbiome in the respiratory system came to light.^27^ With further advances in detection technology, scientists have applied computed tomography (CT) scans, PCR, and 16S rRNA sequencing to investigate the lung microbiome.^8,28^ Since 2011, the relationship between various lung diseases and microbiomes has been gradually explored. The presence of a microbiome in COPD patients has been found.^3^ At the same time, Foder et al. found that adult CF is closely related to the microbiome.^29^ Not only are the survival and prognosis of patients with COPD related to lung microorganisms,^30^ but pneumonia^31^ and bronchial diseases^32,33^ also involve an imbalance in pulmonary microorganisms. In 2014, researchers focused on the relationship between lung transplantation and the microbiome, showing that the impact of the lung microbiome on innate and adaptive immune responses is beginning to be explored.^34,35^ With the further elaboration of the concept of the “microbiome”, the focus is no longer limited to bacterial communities but also to fungal and viral communities. For example, Nguyen et al. uncovered the impact and significance of pulmonary fungal communities on respiratory diseases.^36^ In 2016, Segal et al. linked the lung microbiome to HIV.^37^ After that, with the maturation of molecular diagnostic techniques, one could precisely characterize and analyze the distribution of the lung microbiome. Relationships between pneumonia, COPD, CF, and microorganisms were further investigated. At the same time, new research areas, such as those involving tuberculosis and sepsis, have been developed.^16,38^ In 2016, the association between intrinsic immunity and microorganisms in the body was explored.^39^ The same lung microbiome is involved in acute respiratory distress syndrome (ARDS) and hematopoietic stem cell transplantation.^40–42^ In 2019, COVID-19 spread worldwide, and the relationship between coronaviruses and microorganisms also became a hot topic.^43^ Currently, lung cancer has become an emerging area of microbiome research; its development, metastasis, and prognosis are microbiome-related (Fig. 1). Therefore, the lung microbiome has the potential to be a diagnostic and therapeutic target for cancer, which will be further investigated in the future.

## The composition of a healthy lung microbiome

**Fig. 2:**
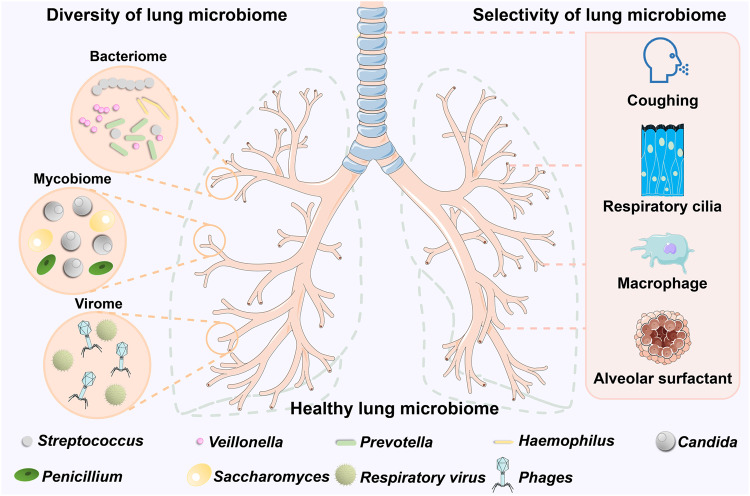
The composition of the healthy lung microbiome. Compared to the rich communities of the intestinal and oropharyngeal microbiomes, the lung microbiome contains fewer resident microorganisms, but this does not mean that it is homogeneous. The lung microbiome is composed of the bacteriome, mycobiome, and virome. Among the bacteriome, *Streptococcus*, *Veillonella*, and *Prevotella* are the most common genera, while *Haemophilus* are unique to the lung as resident inhabitants and are rare in other microbiomes. The mycobiome is less numerous, with *Candida* dominating, followed by *Saccharomyces* and *Penicillium*. *Phages*, on the other hand, dominate the virome in addition to the presence of a small number of respiratory viruses. The lung microbiome has a unique mobility characteristic, which is created by the clearance mechanisms of the respiratory system. Coughing, the movement of respiratory cilia, phagocytosis by macrophages, and alveolar surfactants comprise the clearance mechanisms of the respiratory system, conferring selectivity to the lung microbiome. (Figures are created with Servier Medical Art and exported under a paid subscription.)

The microbiome is the culmination of all the microbes and their gene sequences (including homologous sequences) in a specific habitat at a specific time.^44^ Thus, to investigate the microbiome of the lung, we should investigate the bacterial, fungal, and viral groups (called the “mycobiome” and “virome”) that exist in the lung. Since the composition and dominant community of the lung microbiome vary dynamically with the state of the lung, different diseases may result in different microbial communities. In this section, we focus on the healthy lung microbiome and investigate the microbiome’s specific composition, structure, and functions in terms of bacteria, fungi, and viruses (Fig. 2). Thus allowing us to summarize the microbiological characteristics of healthy lungs for the early detection of respiratory diseases.

### Bacteria (bacteriome)

The core lung microbiome includes *Pseudomonas*, *Streptococcus*, *Proteus*, *Clostridium*, *Haemophilus*, *Veillonella*, and *Porphyromonas*.^3,27,45^ Most of the flora are aerobic or parthenogenetic anaerobic, except for *Clostridium*, *Veillonella*, and *Porphyromonas*, which are specialized anaerobes. There is a part of researchers who think human-associated microorganisms cannot be cultivated in the laboratory. However, a significant percentage of the bacteria and archaea in our microbiota have been cultured.^46^ As laboratory techniques have matured, more species have been successfully cultured, which suggests that microorganisms are not “unculturable“.^47^ Among these bacteria, Firmicutes and Bacteroidetes are the most common phyla, and *Streptococcus, Prevotella*, and *Veillonella* are the most common genera.^48^ Research has indicated that the habitant of the lung microbiome is not permanent but changes dynamically according to the immune response of the body and the migratory movements of URT.^27,49^ Consequently, in contrast to sites such as the skin and gut, which have robust and self-sustaining microbiomes, the lung microbiome may migrate from adjacent sites such as the oropharynx and the URT. The continuous migration of microorganisms between these sites implies that the lung microbiome is in a constant state of flux, with new species being introduced or removed randomly.^48,50,51^

Based on this conjecture, traditional studies have suggested that the species of lung microbes are similar to those in the oropharynx.^3,52^ This may be because the oropharynx and lungs are connected by the URT. However, although the composition of the two regions was similar, the proportions of microbial populations were different, and even the lungs had a unique genus. Comparing BALF and oral fluids in a healthy population shows that the lung microbiome contained different proportions of *Ralstonia*, *Bosea*, *Haemophilus*, *Enterobacteriaceae*, and *Methylobacterium* than the oropharynx. *Ralstonia* and *Bosea* are overrepresented, while *Haemophilus* and *Enterobacteriaceae* are not proportionally abundant in the oropharynx.^53^ In summary, the lung microbiome also has a long-term and self-sustaining bacterial population. After extensive research, we know that the gut microbiota plays a dominant role in regulating gut mucosa development and digestive system maturation.^54–56^ The eubiosis of the lung microbiome provides a clean and safe environment by participating in the immune response and preventing inflammation. By analogy, scientists speculate that the lung microbiota also has the same function of regulating the lung environment and immunity. Individuals lacking lung-specific microbial communities exhibit T helper 17 (Th17)/neutrophil mucosal immune features and have weaker innate immune function, suggesting a potential immunomodulatory mechanism.^57^ In the experiments of Dickson et al., the community composition and bacterial diversity of mouse lungs were negatively correlated with the levels of inflammatory cytokines, including interleukin-1α (IL-1α) and IL-4, showing the influence of the pulmonary bacteriome on inflammation and immunity.^58^

### Fungi (mycobiome)

Fungal analysis usually uses targeted internal transcribed spacer or 18S rRNA genes or shotgun macrogenome sequencing.^59^ Although fungi have been emphasized in recent years,^60,61^ the low fungal biomass in the lungs, the small number of fungal taxa, the difficulty in extracting DNA from fungi, the bias of 18S rRNA gene amplification, and the inconsistency in naming make fungal database annotation unsatisfactory.^62–64^ Compared to the large family of bacteria, the presence and role of fungi are often overlooked.

According to the available studies, the fungal species in healthy lungs are diverse and differ from those in diseased lungs.^29,65,66^ *Ascomycetes* and *Streptomyces* are the most common taxa, followed by *Candida*, *Saccharomyces*, *Penicillium*, *Dictyostelium*, and *Fusarium*.^67,68^ Among them, *Candida spp*. predominate.^66,69,70^ In addition, *Aspergillus*, *Davidiellaceae*, and *Eurotium* are also present.^69^

Unlike bacteria that are directly involved in regulating the pulmonary environment and the organism’s immune response, scientists have found that the lung mycobiome seems to be a cofactor in the host immune response and inflammation.^63^ Accordingly, the pulmonary mycobiome may contribute to decreased lung function and disease progression.^71,72^ Furthermore, the fungal group influences bacterial behavior through different interactions, resulting in positive or negative interactions between members of the lung microbiome.^73–75^ Specifically, fungi and bacteria can produce biofilm structures that protect fungi and/or bacteria from dehydration, drugs, and immunocyte attack. This leads to the development of strains that are multidrug-resistant to antimicrobial agents and capable of spreading.^76,77^

Currently, studies at this stage have begun to provide a promising understanding of fungal-bacterial interactions and their role in the health of organisms and diseases. Recent studies have shown a strong relationship between fungi and cancers. Fungi, although few in number, are prevalent in all major human cancers, and specific fungal community types predict the prognosis of cancers.^78^ In colon cancer, *Candida* not only predicts advanced disease and metastasis but is also associated with diminished cell adhesion. Moreover, the interactions between the bacteriome and mycobiome have been partially investigated. For example, *Candida* was positively correlated with *Lactobacillus* but inversely correlated with *Helicobacter pylori* (*H. pylori*). *Lactobacillus* has been shown to affect the attachment of *H. pylori* and *Candida albicans* to epithelial cells, which may play a role in their colonization.^79^ Fungi are also potential pathogens in the lung, although they seem to play a symbiotic role most of the time; thus, analysis of the mycobiome is of clinical value.^80,81^ By determining the respiratory characteristics of specific fungal groups that can prevent or treat disease, fungal communities may become a target for research in the respiratory system.^65^

### Virus (virome)

The virome can be defined as the sum of all viruses discovered in each environment. The human virome includes all prokaryotic and eukaryotic viruses that exist in the human body. They vary according to position because each location creates a distinct microenvironment. The absence of any conserved regions in viruses similar to bacterial 16S or fungal 18S genes has led to stagnation in studies of the virulence group. With technological developments, scientists have used NGS to effectively probe the composition of the human virome. In humans, the number of viral particles differs depending on the body part.^82^ For example, there are 109 particles/g of virus in gut contents and 108 particles/ml in the oropharynx, nasal cavity, pharynx, and saliva.^83,84^ However, viral particles in the lungs are less abundant than that in the intestine and oropharynx.

Most viral sequences are grouped into the following three main families: *Paramyxoviridae, Picornaviridae*, and *Orthomyxoviridae*.^85^ *Alpha papillomavirus*,^86^ *KI polyomavirus*, *WU polyomavirus*, and *Adenoviridae Masto adenovirus*^87^ are present in the respiratory tract of healthy humans. In addition, a newly identified family of viruses, named *Redondoviridae* for their ring-shaped genome, was identified in the macrogenome sequence of the respiratory tract of healthy and diseased patients.^85,88^ A study of respiratory viromes showed lower viral community complexity in healthy individuals.^89^ For example, in healthy children, the virome consists mainly of members of *Anapoviridae*, with a smaller proportion of human herpesvirus (HHV). In disease-free lungs, the *Anapoviridae* family is the predominant eukaryotic virus, with the occasional detection of *herpesviruses, papillomaviruses, retroviruses*, and other respiratory viruses.^90^

Both symptomatic and asymptomatic individuals carry a variety of eukaryotic viruses. They can regulate health and disease and affect the physiological state of hosts.^91–93^ For example, in the BALF of patients receiving lung transplants, different families of *Anelloviridae* formed nearly 70% of the lung virome, and they were considered pathogenic.^94^ In addition, different viruses cause diseases with varying effects on lung metabolism.^95^ The virome functions differently in the healthy lung than in the disease state, or even the opposite. Studies suggest that viral latency may prove advantageous to the host, as it creates an upregulated basal immune status to control subsequent bacterial infection.^96,97^ Through long-term viral latency, the body continues to produce IFN-γ and activate macrophages. Hence, mice latently infected with murine herpesvirus are resistant to infection by *Listeria monocytogenes*.^98^

Experts have found that phages are plentiful in the lungs, and phage populations vary with the number of bacteria in the host.^86^ Moreover, some believe that a resident core group of 19 phages is present in the human respiratory tract.^86,99^ Phages have a strong and direct influence on bacterial structure. Bacteria can use their prophages to kill associated bacteria or to avoid the excessive growth of certain bacteria using the same ecological niche, which helps bacteria survive and reproduce. At this stage, most of the research has been done on DNA viruses and less on RNA viruses and retroviruses, and our exploration of the pulmonary virome is not yet complete. Future studies may concentrate on the collaboration of viruses with other microorganisms in the lung microbiome. It may unravel the mystery of disease action by clarifying the mechanisms of influence between different organisms.

## The connections of the lung microbiome

Oropharyngeal and gut microbes were studied for a long time before lung microbes were discovered.^100,101^ As mentioned above, unlike the lung microbiome, the oropharyngeal and gut microbiomes are stable and robust, with a profound impact on the physiological and pathological state of the organism. Researchers first speculated that the lung microbiome was the same as the oropharyngeal microbiome due to its location. As study progressed, scientists corrected their view, acknowledging the similarities but also pointing out the differences between these two anatomical sites. However, it is undeniable that the oropharyngeal microbiome has an impact on the production, maintenance, and changes in the pulmonary microbial community. Oral health has been shown to be associated with the risk of developing respiratory diseases.

**Fig. 3:**
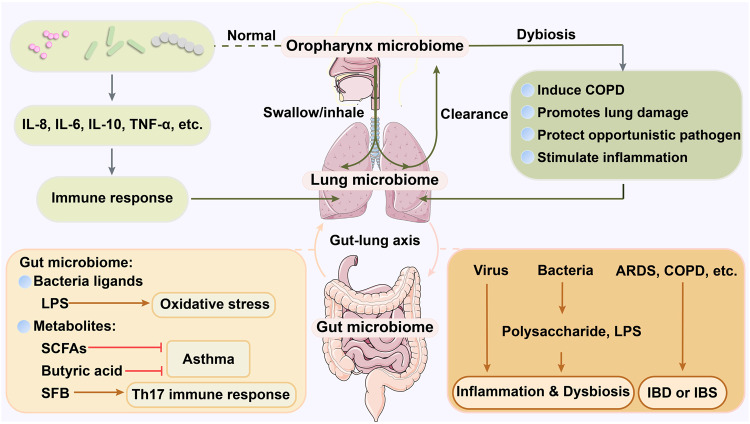
The connection between the lung, oropharynx, and gut microbiomes. The oropharynx and gastrointestinal tract contain abundant and powerful microorganisms that profoundly influence the metabolic activity of the organism, including the lung. Through swallowing or inhalation by the host, the oropharyngeal microbiome is able to migrate. Part of it is repatriated by clearance mechanisms, while other part stays in the lungs and become members of the lung microbiome. Therefore, the lung microbiome is closely, but not identically, related to the oropharyngeal microbiome. The normal state of the oropharyngeal microbiome contributes to the lung immune response by inducing the production of varying levels of cytokines, such as IL-8, IL-6, IL-10, and TNF-α. On the other hand, a dysregulated oropharyngeal microbiome has the ability to stimulate lung inflammation and lead to lung damage. Its internal environment provides protection against opportunistic pathogens that induce COPD. The gut microbiome is linked to the lungs through the gut-lung axis. The gut-lung axis is bidirectional; the gut microbiome influences lung ecology through ligands and metabolites and is involved in the development of lung diseases. For example, LPS may regulate the TLR4/NF-kappaB pathway in the lung immune system, activating oxidative stress in the lung and mediating lung injury. SCFAs and butyric acid can mediate protection from asthma, while SFB can stimulate Th17 immune responses in the lung. In turn, members of the lung microbiome (such as polysaccharides and LPS) can mediate gut dysbiosis. Some viruses can decrease the abundance of the gut microbiome by inducing the production of IFNs in the lung. Moreover, pulmonary diseases, such as COPD and ARD, often accompany the development of chronic gastrointestinal disorders (e.g., IBD and IBS). (Figures are created with Servier Medical Art and exported under a paid subscription.)

Gastrointestinal microorganisms have long been valued for their high complexity and abundance. They not only regulate the state of a healthy organism but are also closely related to different kinds of diseases. Recently, researchers have made the novel discovery that the effects of the gastrointestinal microbiome on the lungs, such as protection against lung disease, may be related to the original inhabitants of the lungs. The lung microbiome also greatly impacts the gut and its microbial communities. In the following sections, we will investigate the association among the oropharyngeal microbiome, the gut microbiome, and the lung microbiome in terms of origin, composition, and function (Fig. 3).

### Oropharynx microbiome

Our knowledge of the oropharyngeal microbiota is significantly more comprehensive than that of the lungs. Similar to the gastrointestinal tract, the oral bacterial microbiome varies among individuals but is stable over time in the absence of external disturbances.^102,103^ In addition, the oral mycobiome and oral virome showed great intra- and interindividual variation.^70,104,105^ This may affect the respiratory and pulmonary microbiota of healthy individuals and patients.

As mentioned above, the oropharyngeal microbiome is similar to the lung microbiome in terms of species; however, there are differences between some colonies. Bacterial communities in healthy lungs were found to have a significant relationship in terms of makeup with the oral cavity, and significant subject differences were noted.^52,106^ In contrast, the nasal microbiome contributes little to the lung microbiome of healthy organisms.^107^ Moreover, researchers found that the number and variety of microbial communities showed continuity from the oral cavity to the lungs.^53,108^ This may provide strong evidence that the lung microbiome originates from the URT and is always in a transient mobile state. Although the bacterial communities in healthy lungs overlap with those in the oral cavity, they are less concentrated, have fewer members and have a different community composition. For example, the researchers detected *Troperyma whipplei* in approximately a quarter of the BALF samples but not in the oral wash. This may be due to the selective nature of the pulmonary environment.^107^ The lungs may select suitable colonies for survival through various rejection mechanisms. It removes microorganisms from the respiratory tract through mucociliary clearance, coughing, and innate and adaptive immunity.^109,110^ Furthermore, the distal alveoli are immersed in alveolar surfactant, which also has inhibitory activity against certain bacterial strains, further creating autonomous selection of the reproductive population and resulting in a sparse lung microbiome.^111^

The oropharyngeal microbiome, as one of the most complex and diverse communities, has a significant impact on the body, and naturally, the lungs are no exception. There is no clear relationship between the oral bacterial community and lung function in healthy organisms, but changes in the oral microbiota may affect lung disease because the lungs are exposed to the oral microbial community through respiratory movements such as breathing and coughing. For example, a healthy mouth and oral bacteria are thought to play a role in COPD.^112^ In addition, certain species of *Veillonella* and *Streptococci*, especially *Veillonella*, can induce the production of varying levels of cytokines, including IL-6, IL-8, IL-10, and tumor necrosis factor-α (TNF-α).^113^ Moreover, respiratory microbiota rich in oral-associated taxa, such as *Rhodobacter* and *Prevotella*, are related to Th17-mediated immune responses in healthy subjects.^31^ These phenomena show that the oral microecosystem is significant in the pathogenesis of COPD based on the stimulation of inflammation and the promotion of lung injury.^17^ Oral short-chain fatty acids (SCFAs) may relieve allergic airway disease,^114,115^ demonstrating that metabolites from the outside or the oral cavity can enter and affect the pulmonary state through the airway and digestive tract. Second, the internal environment of the oral microecosystem also protects against opportunistic respiratory pathogens.^116,117^ More importantly, oral microorganisms enter the lungs through subclinical aspiration and spread across the long bronchial mucosa, directly forming the pulmonary microbial community and directly influencing the growth of pulmonary bacteria.^118–120^

In general, the lung microbiome is close to the oropharyngeal microbiome, and most organisms in the lung are also detected in the oral cavity and URT. Nevertheless, the lung is selective in certain ways. The flora from the URT is reduced through the use of multiple exclusion mechanisms, ultimately retaining a small number of microbial communities. Dysbiosis in the oral cavity may precede or lead to dysbiosis in the lungs and contribute to disease pathogenesis.^53^ The oropharyngeal microbiome is closely related to the lungs, which also leads to experimental errors. BALF often runs the risk of contamination by oral microorganisms, making the experiment somewhat restrictive.^52^ However, the influence of the oropharyngeal microbiome on the lung is still very promising. It may be a useful target for determining lung disease progression and may provide new ideas for clinical disease treatment. Finally, due to the absence of systematic research regarding the pulmonary effects of periodontal and gingival microbiota, studies need to be further systematized.

### Gut microbiome

As the most intensively studied microbial community to date, the composition, structure, and function of the gut microbiome have been well elucidated.^121^ The intestinal flora is mainly composed of the phyla Firmicutes, Bacteroides, Aspergillus, and Actinobacteria, in addition to other bacteria, including *Clostridium*, *Verruciform*, and *Spirochetes*, which occur sporadically. The core gut microbiome includes up to 14 bacterial genera and 150 bacterial “species“.^122–124^ Compared to the gut microbiota, the lung microbiota has lower α-diversity and abundance. By studying how the local microbiome affects immunity at remote locations and how the gut microbiota affects other organs, scientists have coined terms such as the “gut-brain axis” and the “gut-lung axis”. As the name implies, the gut-lung axis refers to the interaction between the gut and the lungs.^125^ The intestinal microbiota consists of thousands of microorganisms that can influence the pulmonary microbiota by producing ligands, metabolites, and immune cells that reach the lungs via the bloodstream to regulate pulmonary immunity. Through these circulating cells and metabolites, the gut microbiome may affect pulmonary immunity directly and possibly the makeup of the pulmonary microbiome.^126^ The pulmonary microbiota is also important in supporting a healthy immune response. Through interactions with epithelial cells and immune cells, it is involved in forming the innate and adaptive immune response in the lung.^127^ In mice, evidence has shown potential connections between the intestinal mucosa and pulmonary mucosa that constitute the gut-lung axis. For instance, polysaccharide-containing airway stimuli alter the gut microbiome, suggesting that the gut-lung axis is able to function in both directions.^128^

The gut microbiome can affect lung function through both immune and metabolic routes. Substantial evidence has proved the key role that the gut microbiome plays in abnormal immune responses, such as in asthma. For instance, in infants and babies, the existence of pathogenic bacteria in the lungs and intestines is related to the subsequent onset of allergic asthma. Depner and colleagues investigated gut microbial changes in newborns from 2 to 12 months and examined the relationship between the gut flora and allergic asthma.^129^ The gut microbiota, via lipopolysaccharide (LPS), may modulate the TLR4/NF-kappaB pathway in the pulmonary immune system, increase oxidative stress and mediate injury in the lung by modulating the intestinal barrier.^130^ Segmented filamentous bacteria in the intestine stimulate Th17 responses in mouse lungs and protect them from *Streptococcus* pneumoniae infection and lethality.^131^ Moreover, parenteral bacille Calmette-Guérin transmission through mycobacterial dissemination induces time-dependent changes in barrier function, microbial metabolites, and the gut microbiome. These intestinal alterations affect subsequent changes in circulating and pulmonary metabolites, resulting in the induction of memory macrophages and innate immunity in the lungs.^132^ It has been shown that some metabolites of intestinal microorganisms, such as SCFAs, can mediate protection against neonatal asthma.^114,133,134^ Moreover, bacterial communities that have the potential to produce butyric acids, such as *Roseburia* and *Coprococcus*, also contribute to asthma protection.^135,136^ Similarly, enteric bacteria found in the BALF have been characterized in ARDS.^16,137,138^

The gut-lung axis is bidirectional, which means that the lung can affect gut homeostasis. A study reported that a nonabsorbable tracer appeared in the gastrointestinal tract of mice shortly after nasal injection.^139^ Intratracheal injection of LPS not only destroyed the respiratory microbiota but also led to the transfer of respiratory bacteria belonging to *Clostridium* into the bloodstream. Then, it affected the intestinal microbiota within 24 h, significantly increasing the total bacterial load.^139^ Influenza virus infection in the airway can lead to gut dysbiosis and drive the lungs to produce interferons (IFNs). For example, the influenza A virus induces depletion of the gut microbiota, disruption of mucus layer integrity, and increased levels of antimicrobial peptides in Paneth cells.^140^ In addition, IFNs produced in the respiratory tract exhibit antibacterial activity and amplify inflammatory responses in the intestinal tract.^141^ Chronic lung diseases such as asthma and COPD often occur in conjunction with chronic gastrointestinal tract disorders, such as inflammatory bowel disease (IBD) or irritable bowel syndrome (IBS).^142,143^ Patients with IBD and IBS also have a certain chance of developing lung disease.^144^ Surveys have shown that intestinal mucosal function and structure are altered in asthmatic patients, while intestinal permeability is usually increased in COPD patients, reflecting the close connection between the intestinal and pulmonary axes.^145^

## Impact on innate and adaptive immune responses

**Fig. 4:**
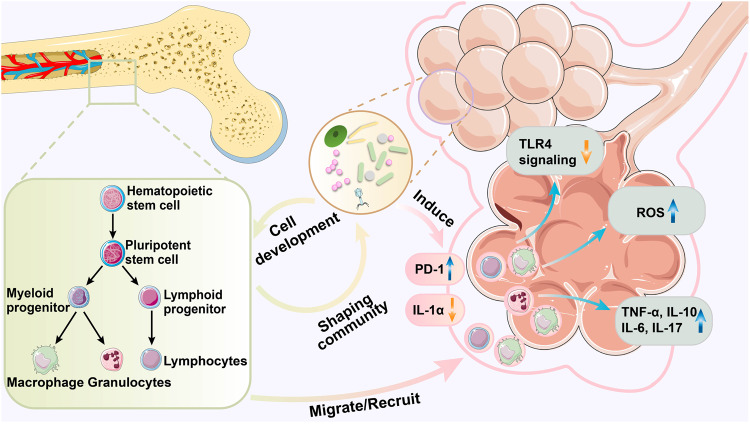
Impact of the lung microbiome on the immune response. The commensal bacteria of the organism are necessary for the maintenance of immune homeostasis. Therefore, alterations in the lung microbiome may contribute to the activation or suppression of immune responses. The microbiota can be detected in the fetal respiratory tract in the early stages of life. As the host grows, both the microbiome and the innate immune system evolve and refine. The microbiome promotes the growth of myeloid cells in the bone marrow, which differentiate to produce immune cells and greatly influence the host’s susceptibility to disease. The innate immune system also plays a significant role in regulating the composition and changes in the microbial community, and microbiome dysregulation occurs in the absence of genes related to innate immunity. Some immune cells migrate or are recruited into the lungs and colonize there, shaping the innate and adaptive immune responses in the lungs. The lung microbiome can participate in innate and adaptive immunity by upregulating the expression of PD-1 while downregulating that of IL-1α. Significantly, the lung microbiome may promote antimicrobial activity by macrophages via ROS, induce immune cells to produce cytokines such as TNF-α, IL-6, IL-10, and IL-17, or inhibit TLR4 signaling. The adaptive immunity response in the lung determines the progression of the disease and thus affects the ecological balance of the microbiome. (Figures are created with Servier Medical Art and exported under a paid subscription.)

Commensal microorganisms in the lungs and gut are essential to the regular development of immune homeostasis. Microbiota dysbiosis, such as changes in the structure, quantity, and variety of bacteria, increases the susceptibility of the host to infection by various pathogens, exacerbates gut and brain autoimmunity and inflammation, induces diverse methods of metabolic disorders, and fosters the progression of neurological diseases.^146,147^ Similarly, interactions between commensal microbes and immune barriers (e.g., gastrointestinal mucosa and urethra) have been formerly identified in diverse tissues.^148^ Thus, microbial dysbiosis can result in irregular inflammatory responses, such as episodes of bronchopulmonary dysplasia (BPD).^149^ Existing studies show that the lung microbiome is important for both innate and adaptive immunity (Fig. 4).

### Innate immune response

The innate immune response is the initial bodily defense mechanism against pathogens. This signaling can be activated by particles, toxins, allergens, microbes, and endogenous debris (such as dead cells) from the ambient air.^150^ Recognition of microorganisms by the innate immune system initiates a signaling cascade downstream of pattern recognition receptors (PRRs) that trigger an immune response.^151,152^ The airway of the newborn is affected by the amniotic fluid, placenta, and vagina during pregnancy and develops its microbiota as early as birth.^153^ These microorganisms are involved in innate immunity and related responses.^154^

Most of the studies describe the airway microbiota present in the early stages of life, which predominantly includes *Staphylococcus* and *Ureaplasma*.^155,156^ The pulmonary microbiome advances during the initial weeks or months after the infant’s birth. The colonization time of microorganisms is controversial. Mourani et al. noticed that during the first 72 h of life, 2 of 10 tracheal aspirates from intubated preterm infants contained detectable microbial DNA, while all samples from this neonate were detectable on the 7th day.^157^ Nevertheless, Lohmann et al. reported that in 25 preterm infants, microbial DNA was detected in all tracheal aspirates inhaled instantly after intubation on the first day at birth.^158^ Moreover, the external environment, such as contamination of the placenta sample, cannot be ignored.^159^ The gut microbiome stabilizes through the first 3 years of life,^160^ but the precise time needed for the respiratory microbiome to fully mature remains to be determined. This is because the respiratory microbiome evolves during the initial years of life.^161,162^ Early lung microbiome colonization profoundly influences the progression of respiratory diseases and the action of the immune system later in life.^163^

Host-microbe interactions influence different aspects of the development of the body’s immune system and contribute to immune maturation, immune tolerance, and immune responses.^164,165^ In the absence of microbiota, myeloid cell differentiation in the bone marrow is reduced, leading to delayed clearance of general bacterial infections.^166^ The effect of the microbiome on the recruitment and gene expression of tissue-resident myeloid cells is accomplished primarily by the regulation of local metabolites and tissue mediators. Microbial PRR ligands can affect circulating granulocytes. Moreover, myelopoiesis is reduced in the bone marrow with the lack of commensal bacteria and their microbial products in the blood. Such alterations in bone marrow greatly affect the susceptibility of the host to various diseases, such as allergies and asthma.^167,168^ In addition, the colonization of symbiotic colonies during the neonatal period in mice reduces CpG methylation in the gene encoding CXCL16, thus protecting mice from the enhanced mucosal accumulation of invariant natural killer T (iNKT) cells in the lung. Moreover, microbial colonization-established mucosal iNKT cell tolerance is critical for protection against the pathogenesis of intestinal inflammation and allergic asthma.^169^ Moreover, in the absence of the commensal microbiota, the antimicrobial activity of alveolar macrophages via reactive oxygen species is compromised, thereby jeopardizing the physiological clearance of potentially pathogenic bacteria.^170^ Certain microbes, such as *S. pneumoniae*, promote a broad intrinsic response in the respiratory tract, support clearance of pathogens, and enhance host survival during infection by signaling the axis of IL-17 and granulocyte-macrophage colony-stimulating factor.^171^ Lung epithelial cells, macrophages, and dendritic cells (DCs) have diverse receptors to sense microorganisms. These include microbial PRR ligands, TLR, and NOD-like receptors.^172^ Epithelial cells activate DCs by translocating microbes.^173^ Below alveolar epithelial cells, DCs present processed antigens to different T-cell subpopulations, thereby activating adaptive immune responses.^174^

Changes in bacterial diversity similarly modulate molecules such as programmed death-ligand 1 (PD-L1) and various innate and adaptive immune populations. For example, during the first two weeks of life, a change in the dominant bacterial phylum from Firmicutes and γ-Proteobacteria to Bacteroides promotes transient PD-L1 expression and modulates general aeroallergen responses and regulatory T-cell (Treg cell) activation.^175^ In a mouse model, the pulmonary microbiota affected host immunity at the focal level.^58^ The concentrations of two significant inflammatory cytokines (IL-4 and IL-1α) were linked to the variety and inhabitant structure of the lung microbiome. In healthy mice, IL-1α is one of the key cytokines for lung innate immune activity against bacteria and is negatively correlated with the variety of bacterial communities and the existence of pathogenic bacteria. Moreover, pulmonary concentrations of these inflammatory cytokines were more closely related to changes in the lung microbiome than to concentrations in the distal intestine or oral cavity.^58^ A dysfunctional lung microbiome encourages the development of disease, while symbiotic bacteria under normal conditions help maintain the body’s health. Symbiotic bacteria in the URT defend against influenza virus infection in mice through the polarization of M2 macrophages and the secretion of anti-inflammatory mediators such as IL-10 and transforming growth factor-β (TGF-β).^176^ Moreover, microbiota-produced metabolites are also associated with the immune response. For instance, exposure of dendritic cells (DCs) to *P. aeruginosa* can induce the production of IL-6, IL-10, and TNF-α by generating high concentrations of putrescine.^177^ In a mouse model, in comparison to the mouse group given salt solution, *Lactobacillus rhamnosus* administration dramatically decreased pulmonary metastasis,^178^ and intranasal administration of rhamnolipid reduced IL-6 levels in the lungs and protected against influenza virus infection.^179^

Models from both humans and mice show the important role of the innate immune response in modulating microbiota variation, composition, and individual differences.^180^ In several innate immunodeficiency mouse models, such as mice lacking the Nod2 gene,^181^ the Nlrp6 gene^182^ or the Tlr5^183^ gene, a biological disorder was found. Consequently, innate immunity promotes the development of beneficial components of the microbiome and is involved in the maintenance of microbial ecological balance. In addition, after the injection of lipopolysaccharide into experimental rats, significant dynamic changes in the diversity, makeup, and function of the lung microbiome occurred with the fluctuation of systemic cytokine levels and the onset and resolution of pulmonary edema. It was also observed that pulmonary microbiota, such as *Curtobacterium*, were related to the hematological percentage of IL-6, IL-10, TNF-α, and neutrophils.^184^ Epithelial cells are linked to multiple mechanisms of interaction with the intrapulmonary microbiota and act as a permeability barrier, sensing microorganisms and responding to their presence.^185^ In the lower respiratory tract, by providing a strong barrier, the airway epithelium serves as the primary line of defense against potentially harmful environmental irritants. It is the first site of interplay with inhaled compounds and is designed to promote the effective clearance of particles and microorganisms by mucus cilia.^186^ In chronic lung diseases, increased mucus production by epithelial cells facilities the growth of bacteria and causes low oxygen concentrations and high-temperature zones, which promote the selectivity and stability of specific bacteria.^187^ Although the functions of epithelial cells in antibacterial and antiviral immunity are well established, little information has been reported on the effect of respiratory epithelial cells on the mycobiome.

### Adaptive immune response

The adaptive immune response includes specific cells (cellular immunity) and immunoglobulins (humoral immunity). This response is dynamic and depends on the exposure of the organism to exogenous substances, as well as the microbial components, metabolites, and local microenvironment.^188^ Using the gut microbiome as a reference, DCs in the gut encounter the resident microbiota. Microbes generate signals that lead to changes in the phenotype of DCs. These DCs not only relocate to the mesenteric lymph nodes, stimulating the creation of regulatory cytokines but also present bacterial-derived antigens to T cells, resulting in their activation.^189^ In addition, activated T cells can access the airway mucosa, where they facilitate anti-inflammatory and protective responses.^190,191^ Due to the close connection between the gut and lung microbiomes, we also propose a strong association between adaptive immunity and the lung microbiome from the perspective of adaptive immunity-associated cells.

As mentioned, enrichment of the oropharyngeal microbiome in the lung, such as enrichment of *Veillonella* and *Revotella*, was correlated with phenotypes of inflammation, including increased Th17 lymphocyte levels, increased inflammatory cytokine expression, and reduced expression of the inflammatory cytokine TLR4 in alveolar macrophages.^57^ In mice, neutrophil infiltration, high levels of IL-6 and TNF-α, and moderate levels of CD4^+^ T-cell-derived IFN-γ and IL-17 were related to *Proteobacterium catarrhalis* infection. For instance, the inhalation of oral commensals in healthy mice induces a prolonged immune response. This includes CD4^+^ and CD8^+^ T-cell activation, Th17 and γδ T-cell recruitment, and other counterregulatory immune responses, including increased Treg cells and increased immune checkpoint inhibitor markers on T cells.^192^ Certain pulmonary microorganisms, including *Staphylococcus*, produce SCFAs to regulate changes in oral microorganisms.^193^ In the epithelial lining of immunocompromised patients, SCFA production is correlated with increased levels of *Mycobacterium tuberculosis* (*M. tuberculosis*) antigen-induced Treg cells.^193,194^ Tryptophan produced from lung fungi is converted to kynurenine by host indoleamine 2,3-dioxygenase, which causes an increase in Treg cells and a downregulation of Th17-mediated mucosal inflammation.^195^ Respiratory viruses can modulate host adaptive immune responses, such by suppressing Th17-induced production of antimicrobial peptides, and *influenza A* promotes *S. aureus* colonization and infection.^196^ Moreover, *Sendai virus* infection in mice is associated with IL-13-dependent NKT cell activation and follow-up airway hyperreactivity.^197^ Likewise, impaired Treg cell function in mice is caused by early infection with *respiratory syncytial virus*.^198^ Bacteria in lung cancer are characterized by a decline in α-diversity and an increase in total bacterial load. The exact outcome of changed microbial diversity has not been elucidated, but studies have previously shown that an increase in α-diversity is often associated with improved survival and therapeutic consequences in several cancers (e.g., cervical cancer) by increasing the tumor infiltration of CD4+ lymphocytes as well as activated subsets of CD4 cells expressing ki67+ and CD69+.^199^ The growth of lung tumors is related to an increase in the number of bacteria and changes in bacterial composition in the airway. Such a dysregulated native microbiome triggers myeloid differentiation factor 88 (Myd88)-dependent production of IL-1 and IL-23 and induces the activation and proliferation of pulmonary resident Vγ6 + Vδ1 + γδ T cells.^200^ The microbiota takes advantage of the pulmonary microenvironment of immunity to foster neoplasm growth and disease development.

Acquired immunodeficiency syndrome (AIDS) pneumonia patients show a higher microbiota diversity than AIDS-free pneumonia patients.^201^ In a case of 60 Ugandan AIDS patients who had their pneumonia treated with antimicrobial therapy, those with decreased airway bacterial diversity showed an increased bacterial load and increased expression of matrix metalloproteinase (MMP)−9 and pro-inflammatory TNF-α.^202^ In addition, the differences in the lower respiratory groups of patients with advanced human immunodeficiency virus (HIV) are much greater than those of healthy individuals. These studies demonstrate that the composition of the respiratory tract microbiota correlates with the immune response. In addition, *Troph eryma whipplei*, the causative agent of Whipple’s disease, is a common bacterium in the lungs of people living with HIV, especially smokers.^203^

## Lung microbiome and respiratory diseases

We have shown that a healthy lung microbiome is transient and is influenced by adjacent body parts and the external environment. Unlike the oropharynx and gastrointestinal tract, the pulmonary microbiome is not an actively colonizing and stationary community. In contrast, in respiratory disease, the microbiota is much more likely to be long-lasting and reside in the respiratory tract and lungs. The change in the status of the pulmonary microbiome from eubiosis to dysbiosis is associated with disease progression.^204,205^ However, the causal relationship between its dysregulation and disease onset remains to be explored. For instance, altered pathophysiology of lung structures and damaged mucus clearance mechanisms may lead to dysbiosis of the microbiome. However, dysregulation of the microbiome may play a pathogenic role in disease by upregulating inflammatory signals or interfering with cytokine production.^206,207^ Thus, the presence of the pulmonary microbiome is a new direction for studying pathogenesis and disease progression. We concisely summarize the association between the pulmonary microbiome and different lung diseases, mainly focusing on the effect of the pulmonary microbiome on COVID-19 and lung cancer (Table 1).

**Table 1:** Clinical trials in lung diseases involving the microbiome

Disease	Biospecimen	Dominant microorganism	Increased microorganism	Related symptoms	Related therapy	Ref.
Asthma	Induced sputum, nasal swab	*Haemophilus*, *Moraxella,* and *Neisseriaceae*	*Haemophilus, Staphylococcus, Pseudomonas,* and *Actinomyces*	Airway mucosa, inflammation, and heterogeneity	Antibiotic therapy, Oral Probiotic, Interleukin (IL)− 4R-alpha Inhibition, and biotherapy	^211–213^
COPD	Sputum, oropharingeal swab, bronchial brush and BALF	*Pseudomonas, Streptococcus, Prevotella and Haemophilus*	*Pseudomonas aeruginosa (P*. *aeruginosa), Lactobacillus, Proteobacteria*, and *Haemophilus*	Emphysema destruction, fine bronchial, and alveolar tissue remodeling	Inhaled corticosteroids	^12–14,213,221,440^
CF	Sputum, throat swab, and nasal samples	*P. aeruginosa, Staphylococcus aureus*, *Burkholderia cepacia*, and *Haemophilus influenzae* (H. influenzae)	*Candida albicans, Stenotrophomonas*, and *Burkholderia cepacia*	Pulmonary deterioration, inflammation, fibrosis, and dysbiosis	Inhaled and systemic antibiotics, cftr modifiers, proton pump Inhibitors, enzymes, nutritional habits	^228,238^
Pulmonary fibrosis	Upper airway swabs and BALF	*Haemophilus, Veillonella, Prevotella* and *Neisseiria*	*Streptococcus, staphylococcal, Actinomyces,* and *Veillonella*	Lung structure destruction, inflammation, and fibrosis	Antibiotic and hormone therapy	^243–245^
Bronchiectasis	Induced sputum and upper airway samples.	*H. influenzae, P. aeruginosa*	*P. aeruginosa* and *Streptococcus pneumoniae*	Permanent enlargement and reduced mucociliary clearance	Antibiotic therapy	^257,258,260^
Pneumonia	Orotracheal secretion and BALF	*Prevotella*, *Streptococcus*, *Clostridium*, *Roseburia*, and *Veillonella*	*Moraxella*, *Streptococcus*, and *Haemophilus*	Increased susceptibility of individuals to pneumonia and inflammation	Probiotic and antibiotic prophylaxis	^48,57^
Lung cancer	Nasal swabs, buccal swabs, lung lesion tissue, and BALF	*Prevotella, Bifidobacterium, Acinetobacter and Ruminococcus*	*Granulicatella, Abiotrophia, Streptococcus* and *Staphylococcus*	changed neoplastic microenvironment and the activity of tumor-infiltrating immune cell	Immune Checkpoint Inhibitor Therapy and antibiotic therapy	^24,431,441^
ALI/ARDS	Whole blood, plasma, BAL fluid, endotracheal aspirate, oral/nasal swab	*Bacteroides*	*Bacteroides, Enterococcus*, and *Lachnospiraceae*	Damage of alveolar endothelium and epithelial barrier, inflammatory cells accumulation, and pulmonary edema onset	Noninvasive ventilation, and Inhaled corticosteroids	^16,442^

### Asthma

Asthma is characterized by abnormal airway mucosa, inflammation, and intermittent wheezing.^208,209^ The complex interactions between the immune system, the lung, the gut microbiome, and the airways, as well as the ecological dysregulation of the microbiome, may be critical factors in asthma development, which may contribute to the heterogeneity of asthma.^210^ Dysbiosis of the microbiome is a basis for the pathogenesis of asthma.^126^ It features an increase in pathogenic communities, such as *Haemophilus* and *Staphylococcus*, as well as *Actinomyces*,^211^ accompanied by a decrease in the number of concomitant commensal bacteria (e.g., *Prevotella* and *Veillonella*).^212,213^ In addition, *Pseudomonas* is a pathogen in many patients, more commonly in patients with severe asthma, but it is almost undetectable in normal lungs.^214^ 16S rRNA analysis of the tracheal microbiome revealed high concentrations of *Haemophilus*, *Fusobacterium*, *Neisseriaceae*, *Sphingomonas*, and *Porphyromonas* in patients hospitalized with atopic asthma and low levels of *Bacteroides* and *Lactobacillus*.^215^ These asthmatic lung microbiotas increase the predictive potential of butyrate and propionate metabolism, which may be helpful for the development of atopic asthma by restricting the bioavailability of SCFAs or increasing the metabolism of SCFAs.^114,215^ The vicious cycle of lung flora dysbiosis, resulting in a higher level of lung inflammation and imbalanced immunity, contributes to the development of allergic asthma and the diverse characteristics of severe asthma. For instance, allergic asthma is triggered by the activation of the body’s innate or acquired immunity through changes in the composition of the bacterial wall or bacterial products in the airway.^216,217^ In addition, the lung microbiome induces chronic inflammatory processes by activating Th2 and other pathways that may exacerbate asthma progression. This inflammatory process may promote specific bacterial colonies, which in turn may lead to further microbial dysbiosis. Finally, certain pathogenic bacteria may affect the immune cell response to drug therapy by influencing the activity of pathogenic factors such as mitogen-activated kinase phosphatase-1.^218^

In general, asthma is characterized by an increase in pathogens of the pulmonary microbiome, which leads to recurrent inflammation and the stimulation, or even exacerbation, of the body’s immune dysfunction. Based on the interaction between asthma and the lung microbiome, more experiments are required to uncover the relationship between the lung microbiome and allergic diseases.

### COPD

COPD is a heterogeneous disease characterized by inflammation-driven bronchitis, emphysema, fixed airflow obstruction, and impaired lung function.^219,220^ The microbiota in COPD patients is significantly different from that in controls. Multiple bacteria are often present in COPD, including potential respiratory pathogens.^221^ In addition, the number of opportunistic pathogenic bacteria, such as *Pseudomonas aeruginosa* (*P*. *aeruginosa*) and *Lactobacillus*, increases with increasing airflow restriction.^221^ We compared the microbiota of 5 COPD patients with 11 asthmatic patients and 8 healthy subjects. It was found that pathogenic Proteobacteria, particularly *Haemophilus*, were increased in asthma and COPD patients.^12,13^ In contrast, *Bacteroides*, especially *Prevotella*, rarely appeared in patients with asthma and COPD.^14,213^

Microbial diversity, which is associated with emphysema destruction, fine bronchial and alveolar tissue remodeling, and CD4^+^ T-cell infiltration of tissues, decreased in the lung microbiome. The emergence of a host immune response to the microbiome also contributes to the pathogenesis of COPD.^222^ In detail, the chronic airway inflammation in COPD is related to a γ-proteobacteria-dominated microbiota.^205^ *Proteobacteria* and *Actinomycetes* are correlated with immune cell infiltration in the lung tissue, which includes neutrophils, eosinophils, and B cells.^222,223^ Compared to healthy subjects, samples from COPD patients showed moderate biocomplexity and specific pathogenic features.^224^ In addition, pulmonary bacteria and their metabolites were linked to clinical outcomes in mild COPD. Some bacterial metabolites, such as adenosine, 5’-methylthioadenosine, sialic acid, tyrosine, and glutathione, are associated with better COPD prognosis.^57,225^

At present, there are relatively few studies on the relationship between fungi and viruses and COPD. The lung mycobiome promotes sensitization in COPD bronchiectasis,^226^ but the involvement of the virome is unclear.

### CF

CF is mainly caused by mutations in the cystic fibrosis transmembrane conductance regulator gene.^227^ In the initial stage, the microbiota of patients with cystic fibrosis is mainly *P. aeruginosa*, *Haemophilus influenzae* (*H. influenzae*), *Staphylococcus aureus* (*S. aureus*), *Burkholderia cepacia*, and *Stenotrophomonas maltophilia*.^228^ As the disease progresses (~1–2 years of age) and the oral microbiota becomes abundant (~3–5 years of age), the disease eventually develops into cystic fibrosarcoma.^229,230^

During clinical stabilization, the airway microbiota of CF patients is relatively unchanged. In contrast, structural alterations in the microbiome are relevant to pulmonary deterioration. Daily sampling for patterns of microbiome changes may be useful in predicting and managing the progression of CF.^231^ Both oral predominant and pathogenic predominant microbiomes were associated with increased inflammation and structural changes in the lung features of cystic fibrosis patients.^230^ Several investigations indicate that a subgroup of the CF anaerobic oropharyngeal microbiota may promote the colonization of pathogens such as *P. aeruginosa* by fermenting mucus to produce fatty acids and amino acids, which such pathogens may use as a carbon source.^232^ *P. aeruginosa* can cause chronic infection, which is associated with an increased risk of pulmonary exacerbation (PE) and increased colonization of diverse pathogens, failure to recover lung function after PE, and rapid loss of lung function over time, causing premature death in patients with CF.^233,234^ Some of these characteristics may be related to gene mutations. Some genes, such as quorum sensing regulators involved in the expression of the virulence factor lasR, are common mutation targets. LasR loss-of-function mutations appear to increase tolerance to β-lactam antibiotics and favor growth on certain carbon and nitrogen sources, thus promoting the growth and colonization of pathogenic bacteria. Moreover, increased numbers and diversity of anaerobic bacteria were associated with less severe disease, better lung function and body mass index, and reduced pancreatic insufficiency. For example, *Prevotella* can reduce *P. aeruginosa*-induced proinflammatory responses in bronchial epithelial cells.^235^ Thus, the lung microbiome may play a necessary role in pathogen formation and the inflammatory response. Furthermore, CF also affects the habitat of microbiota and even causes different microbiome changes depending on the cause of the disease. As intermittent *P. aeruginosa* infection occurs in CF without dysbiosis, chronic *P. aeruginosa* infection leads to pulmonary dysbiosis. The overall decrease in α-diversity in patients suffering from chronic *P. aeruginosa* infection, including a decrease in the specificity of *Prevotella*, *Neisseria*, and *Veillonella*, results in a reduction in the diversity of the lung microbiome, along with a change in the dominant population.^236^ The microbiome composition of CF subjects differs significantly from that of healthy patients and exhibits poor diversity. Each patient has a unique population, usually controlled by one or a few major colonizing bacteria and pathogens, such as *Pseudomonas*, *Staphylococcus*, *Stenotrophomonas*, or *Achromobacter*.^224^ Similar to other lung diseases, the deterioration of lung function in CF patients is also inversely correlated with microbial diversity. When lung function declines, the lung microbiome becomes dominated by CF pathogens. The lung microbiome is therefore used clinically as an indicator of disease progression.^237^

Increasing evidence suggests that fungal and viral groups play a key role in CF. For example, in patients with CF, *Candida albicans* increased in the lungs and grew together with *P. aeruginosa*.^66,238^ Moreover, *Malassezia* was detected as an abundant community in asthmatic and CF patients but not in controls.^239^ In addition, rhinovirus promotes the colonization and reproduction of cystic fibrosis-associated pathogenic bacteria.^240,241^

Overall, patients with CF have specific pathogens at each stage, which change during the progression of the disease. The molecular mechanisms in CF mediated by the lung microbiome need to be further explored, and its role at the genetic level also deserves attention.

### Pulmonary fibrosis

Pulmonary fibrosis is a chronic, progressive, and lifelong lung disease in which the lungs become damaged and scarred. This kind of lung damage can be caused by many different factors, including silica, fibers, radiation, drugs, and inflammation-related diseases. Idiopathic pulmonary fibrosis (IPF) is a prototype featuring extracellular matrix expansion and lung structure destruction with unknown causative factors.^242^

The most common bacteria in the lungs of IPF patients are *Prevotella*, *Veillonella*, and *Escherichia*.^243^ The number of *Streptococci* in airway microbiome samples in patients with IPF also increased.^243,244^ Notable differences in the composition and dominant species were discovered when comparing the lower respiratory microbiomes of chronic hypersensitivity pneumonitis (CHP) and IPF patients. In IPF, Firmicutes dominated, while *Proteobacteria* accounted for a smaller proportion. At the genus level, the *staphylococcal* load increased in CHP, and the *Actinomyces* and *Veillonella* loads increased in IPF.^245^ *Staphylococcus nepalensis* releases corisin, a peptide considered to be conserved in various *staphylococci*, to trigger the apoptosis of lung epithelial cells. This finding reveals the molecular basis for the elevation of *Staphylococcus* in IPF.^246^ The existence of specific members of *Staphylococcus* and *Streptococcus* is related to the progression of IPF and, in particular, to a poor prognosis of IPF patients.^243^ During bleomycin-induced pulmonary fibrosis in mice, dysregulated lung microbiota, such as *Bacteroides* and *Prevotella* dyregulation, promotes the formation of fibrotic pathogens through IL-17R signaling and drive IL-17B production through their membrane vesicles, thereby promoting lung inflammation and fibrosis.^247^ Notably, the reduction in gut microbiota alone did not affect the pathogenesis of bleomycin-induced pulmonary fibrosis, suggesting that dysbiosis of the lung microbiome may be the key pathogenesis of IPF.

Lung bacterial burden predicts fibrosis progression in IPF patients. Research has shown that the bacterial burden of IPF patients is twice as high as that of healthy people. It is closely related to the rate of decline in lung volume and the risk of death.^244^ Homeostasis of the lung bacterial community correlates with the expression of host defense genes in peripheral blood.^248^ Among them, impairment of host defense and immune signaling is one of the factors influencing the severity of IPF.^249^ Patients with IPF who had progressive disease had significantly higher bacterial loads than those without progressive disease. The decrease in alveolar bacterial diversity was significantly correlated with an increase in the concentration of proinflammatory fibrotic cytokines and growth factors in the alveoli, which are also closely linked to IPF.^250,251^ It is worth mentioning that there is a positive correlation between alveolar IL-6, which has proinflammatory and profibrotic functions, and the relative abundance of Firmicutes. This may explain the acute exacerbation of IPF manifested by diffuse alveolitis and altered lung microbiota.^252^

Studies have shown that immunosuppression is associated with IPF progression, including autoimmune reactions and immune dysregulation.^253,254^ The lung microbiome also plays a nonnegligible role in the immune response to IPF. In IPF patients, increased *Streptococcal* abundance was related to increased nucleotide-binding oligomerization domain (NOD)-like receptor signaling, whereas lymphocytes expressing C-X-C chemokine receptor 3 were closely related to *staphylococci*. Downregulation of some immune response pathways, including the NOD, Toll, and RIG1-like receptor pathways, is associated with shortened progression-free survival (PFS). Ten of the 11 PFS-related pathways were associated with microbial diversity.^39^ A comprehensive analysis of genomic and microbial characteristics revealed a significant host response to more abundant or altered microbial communities, indicating that bacterial communities in the lower respiratory tract might serve as a persistent stimulus for recurrent alveolar injury in IPF.^248^

In summary, microbial-host interactions, which are important factors involved in the development of IPF, deserve further exploration. Moreover, some specific microorganisms (e.g., *Staphylococcus* and *Streptococcus*) have a strong connection with IPF progression, suggesting that more species need to be studied.

### Bronchiectasis

Bronchiectasis is a respiratory disease in which there is permanent enlargement of parts of the lung’s airways, leading to a build-up of excess mucus similar to CF that can make the lungs more vulnerable to infection. Patients with bronchiectasis often develop acute infectious lung deterioration, characterized by fever, sputum and dyspnea.^255^

Sputum specimens from a large number of patients with bronchiectasis showed that Firmicutes and Proteobacteria were associated with severe bronchiectasis.^256^ The most common pathogen is *H. influenzae*, while *P. aeruginosa* and *Streptococcus pneumoniae* (*S. pneumoniae*) are the most common lethal pathogens.^257,258^ After acute exacerbation, the community composition showed little change, which means that some of the dominant flora did not correlate significantly with disease progression.^229,259^ However, a decrease in microbial diversity, especially that associated with *Pseudomonas* dominance, was correlated with a higher risk of bronchiectasis severity, frequency of deterioration, and mortality.^260^ This may be due to a reduction in the relative abundance of other organisms sensitive to macrolides during treatment with macrolides and a decrease in the overall diversity of the microbiota, leading to a higher relative abundance of *Pseudomonas*.^261^ Typically, chronic infection with *P. aeruginosa* is strongly associated with increased rates of disease progression and mortality.^262,263^ *P. aeruginosa*, *Aspergillus fumigatus*, *nontuberculous mycobacteria* (NTM), or a combination of these may contribute to the acceleration of progressive lung injury.^264,265^

Although studies are more limited than those on bacteria, fungi, and viruses have also been proven to be involved in the process of bronchiectasis. For example, *Aspergillus* abundance changed considerably in patients with bronchiectasis compared with healthy controls. Its abundance was linked with the worsening of the condition, suggesting that *Aspergillus* may be an important cause of airway inflammation in some patients.^266^ *Aspergillus fumigatus* and *Aspergillus terreus* predominate in bronchiectasis in Asian and European countries, respectively.^266^ In addition, a study of bronchiectasis in children showed that respiratory viruses, especially *rhinoviruses*, were found in 48% of the subjects.^267^ In research on Chinese patients with acute episodes of bronchiectasis, a significantly higher number of virus-positive samples were found in the acute phase than in the stable phase.^268^

Bronchiectasis is essentially a pathological endpoint that can be approached through numerous diverse routes, including abnormal permanent bronchial dilatation and airway obstruction.^269^ Approximately 50% of patients with bronchiectasis have no apparent or easily identifiable underlying cause.^270^ The incidence of bronchiectasis varies by race.^271,272^ Some scientists believe that bronchiectasis is related to the mucociliary clearance rate of the airways. The reduced clearance allows certain microorganisms to colonize the airway by releasing factors that suppress and destroy the ciliated epithelium. Such colonization behavior contributes to a nonspecific immune response in the organism, which ultimately leads to respiratory damage. This progressive lung injury further weakens the clearance mechanism, creating a vicious cycle.^259^ In a recent study, *Neisseria subflava* cultured from patients with bronchiectasis promoted the loss of epithelial completeness and inflammation in primary epithelial cells.^273^ Infection with neutrophilic airway inflammation is considered to be one of the main contributors to bronchiectasis,^255,274^ while high levels of active neutrophil elastase are correlated with low microbial diversity, especially with *P. aeruginosa* infection.^275^ Moreover, in an adult bronchiectasis cohort, the richness of *Rothiaspecies* was negatively correlated with proinflammatory markers in sputum, such as IL-8 and IL-1β and MMP-1, MMP-8, and MMP-9.^276^

In summary, bronchiectasis is a disease fueled by a vicious cycle of continuous bacterial infections and a process of environmental dysregulation, accompanied by tissue injury and damaged lung function. Nevertheless, we still lack clear insight into the immune-inflammatory pathways that influence this disease, and the relationship between the microbiome and specific mechanisms needs to be further explored.

### Pneumonia

Pneumonia is an inflammatory condition of one or both of the lungs usually caused by bacteria, viruses, or fungi and less commonly by other microorganisms. This disease is one of the most dangerous threats for young children and elderly people and can range in severity from mild to life-threatening.

Enrichment of the lower respiratory microbiota and oral bacteria (e.g., *Prevotella*, *Streptococcus*, *Clostridium, Roseburia*, and *Veillonella*) is correlated with subclinical inflammation.^48,57^ Studies of HIV-infected patients in Uganda and the United States have shown changes in the oral and pulmonary microbiota in antimicrobial-treated HIV-infected patients during acute pneumonia, as evidenced by reduced diversity and imbalance.^202,277^ Using a clustering method for sequencing data from 16S rRNA genes, lower respiratory tract samples from HIV patients with pneumonia can be organized into different groups. One group was dominated by Pseudomonadaceae. The second group is divided into two subclusters rich in Streptococcaceae or Prevotellaceae.^278^ The Pseudomonas-rich microbiome may suppress the virulence of potential pathogens and promote the restoration of the lung microbiome to a healthy state. Conversely, lower respiratory microbiota enriched in Streptococcaceae or Prevotellaceae may favor the persistence and multiplication of pathogens by promoting virulence factors or pushing nutrients into the alveolar cavity.^100^ Even the microbiome of patients with pneumonia varied depending on the clinical course. For example, patients with ventilator-associated pneumonia had a higher bacterial load in the intratracheal aspirate than controls but fewer bacterial species.^279^ In contrast, *Pseudomonas*, *Corynebacterium*, and *Roseburia* were more abundant in patients with pneumonia who were intubated, while *Streptococcus* and *Prevotella* were less abundant than in patients without pneumonia.^280^ In addition, the abundance of Firmicutes and Streptococci decreased in patients with interstitial pneumonia, and the abundance of *Prevotella* and *Veillonella* increased.^281^ After lung transplantation, subjects diagnosed with pneumonia had a reduced diversity of lung microbial communities, which were dominated by *Pseudomonas*, *Staphylococcus*, and *Streptococcus*.^282^ Some of the host’s immune mechanisms may be impaired due to dysregulation of the lower respiratory tract, thus increasing the susceptibility of individuals to pneumonia. For example, SCFAs such as butyric acid directly affect T-cell function by inhibiting the production of INF-γ and IL-17.^194^

The lung microbiome and pneumonia interactions are complex, dynamic, and bidirectional. Animal experiments have effectively demonstrated the relationship between the lung microbiome and the development of pneumonia. For example, sterile direct lung injury in mice resulted in an increase in lung bacterial content and a shift toward the excessive growth of specific colonies. When bacterial communities from such injured lungs were introduced into the lungs of healthy mice, they caused more inflammation and injury than bacteria acquired from uninjured lungs.^283^ Second, changes in the lung microbiota may affect the natural course of pneumonia in diseases such as COPD and CF, and patients with lower α-diversity may have a poorer prognosis and an accelerated rate of deterioration.^284^ Bacterial diversity was reduced in patients with intermediate and advanced COPD compared to those with early COPD. When infected with a new strain or a change in bacterial load, patients usually experience increased inflammation and rapid loss of lung function.^285^ The sudden appearance of bacterial pneumonia is characterized by a potential positive feedback loop; once started, the pro-growth signal is gradually amplified, forming a vicious cycle of homeostatic disturbance and increased inflammation.^100^ In addition, proton pump inhibitors increase the risk of pneumonia by reducing the clearance of gastric microbiota and increasing the migration of bacteria to the lungs.^286^ Early intensive care studies with probiotics have shown that probiotics can reduce the risk of pneumonia and shorten the stay of ventilated patients in the intensive care unit.^287^ It is evident that the microbiome is strongly associated with the prognosis of critically ill patients. The emergence and progression of pneumonia also affects microbiome composition and homeostasis. Regardless of the cause of lung injury, an inflammatory cascade may be triggered, leading to an increase in alveolar catecholamine concentrations.^288^ In turn, it promotes the growth and virulence of selected bacterial community members so that alveolar inflammation persists for a long time.^289,290^

In short, by regulating cytokines and metabolites, the lung microbiome promotes inflammation that results in the progression of pneumonia. This regulatory mechanism is complex and bidirectional, implicating multiple diseases and influencing the composition of the lung microbiome. The interactions between the pulmonary microbiome and pneumonia require thorough study to manage the progression of inflammation and improve the prognosis of pneumonia patients.

### COVID-19

As the ongoing severe acute respiratory syndrome coronavirus 2 (SARS-CoV-2) pandemic has firmly put pulmonary research firmly into the global spotlight, we separate research on the correlation between SARS-CoV-2 and lung microbiomes from pneumonia to highlight the progress in this area. Since the beginning of 2020, COVID-19 has been widely distributed throughout the world. By 2022, numerous variants of COVID-19 have emerged, making diagnosis, treatment, and vaccine development extremely difficult. The role of the lung microbiome in this disease has not been clarified, but studies have found an association between the two. Pulmonary bacterial and fungal flora are associated with nonresolving ARDS in pneumonia, contributing to the heterogeneity of clinical outcomes in ARDS.^43^

From the literary analysis, we can conclude that the modulation of the gut and lung microbiome is promisingly envisaged as an adjuvant for the prevention or treatment of patients with COVID-19 due to the immunomodulatory properties associated with probiotics and prebiotics.^291^ To date, few clinical studies involving the use of probiotics in patients with COVID-19 have been completed, but all have found a reduction in the duration of illness and the severity of symptoms, such as fatigue, olfactory dysfunction, dyspnea, nausea, vomiting, and other gastrointestinal symptoms. Invasive mechanical ventilation is often required in critically ill patients, and the probability of successful extubation is closely related to the microbial load of the lung. Studies have shown that patients with an increased pulmonary microbial burden have a lower probability of recovery from invasive mechanical ventilation and a higher mortality rate.^292,293^ This may be associated with alveolar proinflammatory cytokines, and alterations in the pulmonary microbiota may affect the host immune response and increase alveolar inflammation.^283^ For instance, lung microbiome composition is related to changes in TNF-α, and microbial factors may activate inflammatory bodies, leading to IL-1β release.^294,295^ This experiment demonstrated that lung bacterial and fungal loads were associated with cytokines and alveolar inflammatory markers (e.g., TNF-α, IL-6, IL-1β) involved in the activation of inflammatory vesicles. These markers are associated with the development of ARDS, an important feature of severe COVID-19.^296,297^ Furthermore, the composition of the pulmonary microbial community in patients with COVID-19-associated ARDS was linked with successful extubation but not with specific individual bacteria. This study provides new ideas for the prognosis of COVID-19 and the treatment of critically ill patients.^43^

Last, secondary infection of the epidemic is also a noteworthy issue. The bacterial culture results of Carter et al. showed that secondary bacterial pneumonia is related to a higher lung bacterial load compared to patients with negative BALF cultures.^298^

COVID-19 is primarily caused by respiratory viruses, and the pulmonary microbiome contributes to the development of severe pneumonia by promoting inflammatory responses and regulating cytokines. The specific alterations of the microbiome in COVID-19 need to be further explored for effective treatment and improved patient prognosis.

### Lung cancer

Symbiotic microbiota have emerged as important biomarkers and modulators of oncogenesis and the therapeutic response to cancer. Nevertheless, our current understanding of the cancer microbiota is mainly restricted to the gut microbiota. As one of the mucosal organs with the largest surface area in the body, the lungs are in contact with diverse microorganisms by inhalation, either macro or micro. The lungs are colonized by various microbial communities under both physiological and pathological conditions.^299,300^ Scientists speculate that ecological dysregulation of the lung microbiome may also play an essential role in tumorigenesis at multiple levels. Perhaps by influencing inflammatory, metabolic, or immune pathways involved in cancer development.^301,302^ Although the impact of the microbiome on various cancers has been extensively explored,^303,304^ few studies have examined the interaction between lung cancer and the microbiome. Starting in 2018, researchers focused on the correlation between lung cancer and the microbiome and reported some interesting findings.

Lung cancer accounts for almost a quarter of all cancer deaths.^305^ Early epidemiological data show that bacterial infections are very common in patients with lung cancer. Up to 50–70% of lung cancer patients have pulmonary infections complicating the disease process.^306^ Pulmonary microbiota, including *Chlamydophila pneumoniae* (*C. pneumoniae*), *M. tuberculosis*, and respiratory viruses, may take part in the development and progression of cancer.^307,308^ The lung cancer microbiome exhibited differences in the relative abundance of several genera compared to healthy individuals. For example, *Streptococcus* and *Staphylococcus* levels were notably higher in patients with lung cancer, whereas the abundance of *Streptomyces* and *Streptococcus* was decreased in noncancerous tissues of cancer patients.^24^ By molding the neoplastic microenvironment and regulating the activity of tumor-infiltrating immune cells, ecological dysregulation of the lung microbiota can be a key contributor to lung cancer development.^309,310^ Dysregulation of pulmonary microbiome ecology or destruction of the gut-lung axis can lead to DNA damage, induce genomic instability, and alter host susceptibility to carcinogenic damage, resulting in lung cancer development.^311,312^ Microbiome dysbiosis causes a reduction in symbiotic microorganisms, and inflammation induces growth in bacteria. Bacterial toxins, including cytotoxic necrotizing Factor 1, cytolethal distending toxins, *Bacteroides fragilis* toxins, and bacteria-driven hydrogen sulfide and superoxide radicals, have been determined to trigger DNA damage to induce carcinogenesis.^313–316^ The microbiome and its metabolites also participate in the progression of chronic inflammation, as we described in the previous section.^317^ Stimulating the human lung cancer cell line A549 with products isolated from cancer patient-enriched bacteria was shown to promote the overexpression of genes related to the phosphatidylinositol 3-kinase (PI3K) and extracellular signal-regulated kinase 1/2 signaling pathways.^318^ PI3K pathway activation has been suggested to be a process of carcinogenesis.^207^ Chronic inflammation is both a crucial feature of COPD and a potential driver of lung cancer development.^319^ Moreover, tuberculosis (TB) has been linked to lung cancer according to epidemiological studies.^320–322^ The study showed a significantly increased risk of lung cancer in patients with preexisting TB.^321^ This implies a strong relationship between lung cancer and TB and demonstrates the influence of the lung microbiome on lung carcinogenesis.

Non-small cell lung cancer (NSCLC) is the main form of lung cancer, of which lung adenocarcinoma (LUAD) and lung squamous cell carcinoma (LUSC) are the main subtypes.^318^ Sputum collected from lung cancer patients with LUSC and LUAD is rich in *Veillonella*, *Neisseria*, and phage.^120^ By analyzing the microbial composition of LUAD samples and comparing them with LUSC samples, scientists found differences in microbial composition and abundance between these subtypes.^323,324^ Among them, the presence of MCs in positive LUAD tissues was particularly critical and was linked with reduced CD36 and increased PARP1 levels.^325^ Moreover, the preoperative composition of lower respiratory tract microorganisms may be associated with early NCSLC recurrence because the microbiome of patients with postoperative recurrence differs from that of patients without recurrence.^326^ After comparing the BALF of NSCLC patients and noncancerous controls, they discovered significant differences between bronchoscopy samples and lobectomy samples. NSCLC patients have similar microbial communities to noncancerous subjects. Among them, the relative abundance of several bacterial taxa, such as Actinomycetota, Corynebacteriaceae, Dunaliellaceae, *Corynebacterium*, *Rathayibacter*, and *Halomonas*, was significantly lower.^327^ Scarce species, for example, *Bacteroides pyogenes*, *Lactobacillus rossiae*, *Paenibacillus odorifer*, *Magnetospirillum gryphiswaldense*, *Pseudomonas entomophila*, and the fungus *Chaetomium globosum*, had significantly different abundances between NSCLC patients and noncancerous controls.^328^ These specific species were detected based on the subjects’ age, sex, and smoking status. The results showed that different subjects with different lung segments had different microbial compositions.^24^ Regarding detailed bacterial taxa, it was reported that the genus *Thermus* is more abundant in tumor tissue in patients with advanced disease, and the high levels of *Legionella* in patients who experienced metastasis imply a function for these bacteria in cancer progression.^10^ Another preliminary study paired lung tumor and distal normal tissue samples from the same region of the lung in 19 patients with NCSLC. It was concluded that patients with a higher diversity and abundance of lung microbiota in unaffected lung tissues had shorter disease-free survival and lower relapse-free survival rates.^329^ In conclusion, lung cancer is related to local microbiota dysbiosis, characterized by increased bacterial abundance, decreased α-diversity, and altered bacterial composition. The occurrence of lung cancer makes the lung microbiome of patients differ from that of healthy individuals, and dysregulated microorganisms act on cancer and influence its development and prognostic course.

Dysbiosis of the microbiome may affect not only tumor progression but also clinical therapeutic effects, especially that of immunotherapy.^330^ Patients with NSCLC treated with broad-spectrum antibiotics before immune checkpoint inhibitor therapy have a poor clinical prognosis.^331^ Similarly, airway enrichment before anti-PD-1 therapy was linked to adverse effects in lung cancer, suggesting that underlying resistance to immunotherapy can be ascribed to the lung microbiome.^332–334^ A high bacterial burden in tumors with an increase in inducible nitric oxide synthase expression is a beneficial prognostic factor. In contrast, the combination of a high bacterial load and an increased number of forkhead box protein 3 (FOXP3)-positive cells is a sign of poor prognosis.^335^ Therefore, the microbial burden of the tumor had two conflicting prognostic values, depending on the status of local antitumor immunity. The detection of resident microbial populations in tumors of different origins makes the microbiome a promising diagnostic marker for clinical purposes, and the lung is no exception. Studies have shown that neomycin and aerosolized vancomycin treatment reduce the implantation of lung tumors. According to this research, aerosolized antibiotic treatment resulted in a decrease in IL-10-producing Tregs and an increase in the activation of antitumor NK and T-cell responses, thus alleviating immunosuppression in the tumor microenvironment.^178^ Shannon’s diversity index was significantly lower when comparing the bacterial community of subjects in the emphysema-only group with those of lung cancer patients.^336^ Furthermore, the abundance of the major phylum Proteobacteria was notably lower in lung cancer patients, while the abundance of Firmicutes and Bacteroidetes was higher.^336^ The enrichment and homogeneity of the microbiome in patients with lung cancer were similar to those in patients with benign lung disease, while the functional differences in the microbiome varied by group.^301^ These changes in the lung microbiome may be markers of lung carcinogenesis and have the potential to be vital indicators for monitoring lung cancer. Consequently, sequencing of the airway microbiota may be a new genomic approach for the early detection of lung cancer and an opportunity to predict the risk of cancer development.^337^ Marshall et al. defined and validated a microbial-based classifier that predicts the development of cancer in patients without clinical signs of cancer before diagnosis. Their findings indicate the potential of utilizing lung microbiome analysis as a tool for the early detection of lung cancer.^338^

In addition to the bacteriome, the mycobiome also has an impact on lung cancer. For example, *Blastomyces* is highly prevalent in lung tumor tissue.^339^ However, it has not been clarified whether *Blastomyces* contribute to the lung cancer phenotype or are enriched because of the development of lung cancer. Moreover, enriched tumor-resident *Aspergillus sydowii* (*A. sydowii*) was identified in patients with LUAD, which participates in the progression of lung cancer. By inhibiting cytotoxic T-lymphocyte activity and PD-1 + CD8 + T-cell accumulation, *A. sydowii* promotes lung tumor progression through IL-1β-mediated myeloid-derived suppressor cells expansion and activation.^340^ In addition, the interaction between different inhabitants in the microbiome needs more studies for clarification. The latest research shows that the interaction between fungi and bacteria can trigger inflammatory responses. These inflammatory responses vary depending on the tumor category. Moreover, this interaction is associated with macrophages, showing the relationship between fungi and the body’s immune system.^341^

In conclusion, the lung microbiome is involved in cancer development, progression, treatment, and prognosis. Determining the mechanisms by which the bacterial community interacts with the mycobiome and virome will require further investigation. The relationship between the lung microbiome and cancer needs to be further explored from this perspective.

### Vascular diseases

Microbial ecology shows a significant correlation with the pathology of many vascular diseases. The lung microbiome is considered to play a significant role in the morphogenesis of the respiratory tract,^11,342^ including the pulmonary capillary network.^343^ Vascular endothelial growth factor content is associated with lung microbial abundance, but determining the related mechanism requires more thorough research.^344^

Pulmonary hypertension (PH) is a progressive disease that occurs in the pulmonary vascular system and features persistent vasoconstriction, vast regeneration, and in situ thrombosis.^345^ The lung microbiome has been proven to influence the outbreak and progression of PH, directly or indirectly. For example, as a mechanism driving the pathogenesis of PH, the systemic inflammatory response is related to the microbiome.^16^ In lung cancer patients, the microbiota of the respiratory tract was related to the upregulation of the PI3K pathway, which was usually activated in PH patients.^206^ Activated extracellular signal-regulated kinase (ERK) and PI3K signaling pathways trigger the proliferation of pulmonary artery smooth muscle cells and contribute to PH disease progression.^346^ The relative abundance of microorganisms in PH patients was significantly different from that in controls. Community diversity values were notably lower in PH patients than in reference subjects. The pharyngeal microbiota of the two groups of patients also showed different characteristics. In patients with PH, the proportions of *Streptococcus*, *Lautropia*, and *Ralstonia* were significantly higher than those in reference subjects. In contrast, the abundance of *Haemophilus*, *Rothia*, *Granulicatella*, *Capnocytophage*, and *Sccharibacteria* was greater in the controls. At the level of the glyph, the number of Bacteroidetes decreased and the number of Firmicutes increased in the PH group compared to the control group. All these changes in colonization were linked to the progression of PH, such as the upregulation of the PI3K pathway involved in *Streptococcus*. However, the mechanisms of *Lautropia* and *Ralstonia* still need to be explored.

Furthermore, diseases such as pulmonary edema and pulmonary embolism are also associated with microorganisms. For example, periodontal disease is a less common cause of infectious pulmonary embolism (SPE).^347,348^ In PD-SPE patients, a large number of *Actinomycetes* were found in the body, and after treatment with vancomycin and clindamycin, the patients’ lung disease was improved, which proved that *Actinomycetes* may cause PD-SPE.^349^ Moreover, when compared with control mice, ovalbumin (OVA) mice exhibited malignant states, such as pulmonary edema. The inflammatory cell content in BALF was reduced, and the inflammatory response was decreased after supplementing OVA mice with *Lactobacillus*. Meanwhile, TLR2/TLR4 expression levels declined, with improved inflammatory cell infiltration in the airway mucosa, reduced alveolar swelling, and thinner basement membranes. This shows that *Lactobacillus* repressed TLR2/TLR4 expression in OVA mice. *Lactobacillus* supplementation reduces the inflammatory response and suppresses pulmonary edema.^350^

In summary, more research is required on vascular diseases and lung microbes. Both the pathogenic mechanisms of each pathogen and other vascular diseases in addition to PH need more study.

### Bronchitis

Bronchitis is a chronic nonspecific inflammation of the trachea, bronchial mucosa, and surrounding tissues, with symptoms mainly manifesting as pronounced cough, sputum production, shortness of breath, and recurrent respiratory tract infections. Patients with bronchitis of different etiologies contains different microbiomes.

The bacterial biomass and percentage of neutrophils, IL-8, and IL-1β in children with protracted bacterial bronchitis (PBB) were markedly higher than those in the control group. Compared to controls, the BAL microbiome of PBB children was different and divided into four distinct lineages. While some of them are mainly respiratory pathogens, others contain a greater diversity of microbiota, for example, *Prevotella*.^351^ In detail, enrichments of *H. influenzae*, *S. pneumoniae*, *Moraxella catarrhalis* (*M. catarrhalis*), and *S. aureus* were detected among cultured PBB microorganisms.^352^

Neutrophil inflammation in PBB kids is related to microbiome multiplicity. The remarkable correlation between bacterial biomass and inflammatory markers suggests that PBB inflammation is not attributable to a sole pathogenic species.^351^ Bronchitis with features related to *Prevotella* has symptoms similar to those of bronchitis with predominantly pathogenic bacteria. Consequently, it is recognized that bacteria that are not pathogens might also assist in the development of PPB inflammation. This explains the fact that some children with chronic cough and lower respiratory tract inflammation, but without cultured respiratory pathogens, still respond to antibiotic therapy.^353,354^

In addition, the lung microbiome was also strongly linked to the prognosis of bronchitis. In children with PBB, low airway infection with *H. influenzae* increased the rate of dilation of bronchial tubes by 7 times.^355^ Research on children with acute respiratory infections revealed that *M. catarrhalis* was related to children’s coughs that lasted 28 days.^356^ Early research on PBB also indicated the essential contribution of *Neisseria* and *Streptococcus*.^357,358^

The lung microbiome is also an important factor in obliterative bronchiolitis (BO). In a study of BO patients,^359^ all subjects exhibited a smaller respiratory community dominated by *Aspergillus*, and bacterial diversity was temporarily reduced with BO and correlated with neutropenia and antibiotic treatment. In particular, the increase in the number of *Actinomycetes*, *Neisseria*, and *Pseudomonas* was significantly relevant to the resistance to BO.^360,361^ It is noteworthy that with the increase in the richness of Aspergillus, the diversity of the bacterial community and the expression of multidrug resistance genes both increased. Since the structure of the subject’s lung microbiota changes before the onset of inflammation and BO, these findings demonstrate that during the development of BO, host-microbe interactions facilitate the regulation of the immune response and exacerbate changes in inflammation and fibroproliferation.^362^ Moreover, features of the microbiome can be used to assess the prognosis of BO. For example, a gram-positive-rich lung microbiome can predict BO resiliency.^363^

Tracheobronchitis is characterized by high microbial diversity. The microbiome of tracheobronchitis is dominated by Pseudomonas and Staphylococcus and consists of a wider variety of phyla, including Actinomycetes, Firmicutes, Ascomycetes, Bacteroidetes, and Tenericutes.^282^ The elevation of proinflammatory cytokines, such as TNF-α, IL-6, and MIP-1α, which are closely associated with tracheobronchitis, may result from a combination of dominant culture-positive bacteria (e.g., *P. aeruginosa*) and progressively depleting anti-inflammatory bacteria (e.g., *Lactobacillus*).^364^ Compared with normal organisms, IL-17A levels were reduced in patients with tracheobronchitis, but cytokines associated with Treg upregulation (e.g., IL-20^365^ and IP-10^366^) increased. Microorganisms that induce Tregs and their cytokines, such as Bacteroides^367^ and Clostridium,^368^ are significantly increased in tracheobronchitis.

The composition of the lung microbiome is specific in different kinds of bronchitis, which complicates the relationship between the two. The composition of the lung microbiome is specific in patients with different species of bronchitis, which complicates the relationship between the two. However, every coin has two sides. Dynamic changes in the lung microbiome can be an indication to distinguish bronchitis and determine the progression of the disease.

### Sarcoidosis

Sarcoidosis is a granulomatous disease affecting multiple organs and is characterized by characteristic noncaseating granulomas. Several infectious agents have been suggested as possible pathogens of sarcoidosis, including *Mycobacterium* and *Propionibacterium acnes* (*P. acnes*).^369^

The most abundant genera in sarcoidosis BALF were *Streptococcus*, *Prevotella,* and *Veillonella*,^370,371^ which is consistent with previous studies on the compositions of the healthy lung microbiome.^31,57^ The specificity lies in the fact that a taxon composed mainly of patients with sarcoidosis featured a microbiota monopolized by the Erythrobacteraceae family,^370^ as well as *Mycobacterium*^372^ and *Propionibacterium acnes*.^373^ In addition, *Atopobium* and *Clostridium* were abundantly present in the microbiota and associated with sarcoidosis.^374^ When comparing sarcoidosis with interstitial lung disease (LID), the abundance of taxa containing *Haemophilus*, *Stenotrophomonas*, and *Enterobacter* was considerably higher in the ILD group, while the abundance of *Corynebacterium* and *Neisseria* was higher in sarcoidosis.^375^ Moreover, compared to other ILDs, lung microbiome analysis on the basis of 16S RNA gene sequencing in sarcoidosis did not show obvious dysbiosis in patients, which may be related to the unknown pathological mechanism of sarcoidosis.^376^

The exact role of pulmonary microorganisms in the pathogenesis of sarcoidosis is unknown, and there is a suggestion that they may trigger the formation of persistent granulomatous inflammation. This is because the granuloma observed in sarcoidosis has certain histological similarities to granulomatous diseases caused by pathogenic infections, such as leprosy and tuberculosis.^377^ One theory suggests that *Atopobium* and *M. tuberculosis* have underlying mechanistic relevance since they belong to the same bacterial phylum. For instance, exposure to *Mycobacterium* antigens through natural infection or BCG vaccination may induce an autoimmune response in susceptible individuals, leading to sarcoidosis.^378^ These two species may share highly conserved antigens that allow *Atopobium* to induce an immune response similar to that of *M. tuberculosis* in patients with sarcoidosis.^379^ The second possible mechanism is the autoantigen-like reaction triggered by the *Atopobium* antigen described in rheumatoid arthritis (RA).^380^ Furthermore, *Clostridium* is a commensal bacterium of the respiratory and intestinal flora and is more aggressive in inflammatory environments^381^; thus, it could possibly be involved in the inflammatory response of sarcoidosis.

*Propionibacterium acnes* was once the only microorganism isolated from sarcoidosis specimens by bacterial culture.^382^ *P. acnes* has been found in granulomas and inflammatory cells of myocardial tissue,^383^ in preretinal granulomas from patients with sarcoidosis-associated uveitis,^384^ and in many other granuloma specimens.^385,386^ *P. acnes* was found in the BALF of most patients with sarcoidosis, and its presence was correlated with the activity of the disease. The existence of *P. acnes* was found in formalin-fixed paraffin-embedded (FFPE) sections of patients from various countries but was rarely detected in tuberculosis and lung cancer patients.^387,388^ Since Treg cells infiltrate heavily in patients with sarcoidosis, accompanied by a high level of associated cytokines, such as IL-10 and TGF-β, *P. acnes* may induce Treg cells by releasing propionic acid, resulting in the formation of granulomas in sarcoidosis.^389^

In short, sarcoidosis is affected by the microbiome in terms of the immune response. However, the exact role that the lung microbiome plays in pathogenesis needs to be explored.

### Acute lung injury (ALI)/ARDS

ALI/ARDS is a common clinical critical illness with a rapid onset and high mortality rate and is one of the major causes of death in critically ill patients.^390^ Its onset is closely related to sepsis, and the mortality rate of ARDS caused by sepsis is higher than that of ARDS caused by other factors.^391^

Sepsis-associated ALI/ARDS contributes to alterations in the lung microbiota in mouse models and patients. The etiology of pulmonary dysbiosis in patients with sepsis is complex and includes endogenous factors (e.g., hypoxia and ischemia‒reperfusion injury) and exogenous factors (e.g., tracheal intubation, pulmonary mechanical ventilation, and antibiotics). ALI is characterized by damage to the alveolar endothelium and epithelial barrier, the accumulation of inflammatory cells, and the onset of pulmonary edema.^392^ By injecting LPS into rats, several experiments have revealed significant dynamic changes in the diversity, composition, and function of the lung microbiota at different time points in response to fluctuations in systemic cytokine levels and the onset and regression of pulmonary edema. In detail, the bacterial DNA loading increased in BALF.^283,393^ In contrast, community complexity as measured by the Shannon Diversity Index was significantly decreased, and α-diversity was significantly decreased.^184,394^

The mechanism of sepsis-induced ALI/ARDS is complex and multifactorial, involving the release of inflammatory cytokines and disruption of the pulmonary microvascular barrier. Transient expression of IL-1β induces acute lung injury and chronic lung repair, leading to pulmonary fibrosis. High expression of IL-1β is accompanied by elevated levels of the local inflammatory factors IL-6 and TNF-α and a strong acute inflammatory tissue response with signs of tissue damage. In terms of lung microbiota function, the abundance of proteins in four signaling pathways, the Wnt, Notch, chronic myeloid leukemia signaling pathway, and mitogen-activated protein kinase signaling pathway-yeast (KO04011), was significantly negatively correlated with serum IL-1β and IL-10 levels, suggesting an association between lung microbiota and the pathogenesis of ALI/ARDS.^184^

Disruption of epithelial and/or endothelial barrier function distinguishes ALI/ARDS from pneumonia. Severe hypoxemia is relatively rare in patients with pneumonia. In contrast, severe hypoxemia is a defining feature of ARDS and is thought to be a consequence of loss of alveolar-capillary barrier function.^395^ Similar mechanisms of cellular infection, invasion, and lysis caused by secreted factors of respiratory microbes may disrupt the integrity of the endothelial cell layer. For example, influenza viruses replicate in ciliated respiratory epithelial cells as well as type I and type II alveolar epithelial cells, disrupting the epithelial barrier and inhibiting alveolar cells from maintaining surface-active substances, which upsets the balance.^396,397^ The balance of epithelial injury and epithelial repair is affected by the lung microbiota and the host immune responses, which are central regulators of the development of ALI/ARDS.

In short, ALI/ARDS has a complex pathogenesis, including the disruption of epithelial and endothelial barriers and the dysbiosis of inflammatory cytokines. Both are closely related to the lung microbiome. Furthermore, cellular damage in ALI/ARDS occurs mainly through caspase-dependent pathways, causing cellular pyroptosis. The association of the lung microbiome with this pathway requires further study and this pathway may be an emerging therapeutic target.

### Other diseases

Apart from the diseases mentioned above, other diseases of the lung are also linked to microorganisms but are less studied. We have summarized them briefly in this section.

Long-term administration of the anti-inflammatory drug roflumilast to mice with secretory immunoglobulin A (SIgA) immunodeficiency due to polymeric immunoglobulin receptor (pIgR) deficiency prevents progressive tracheal wall remodeling and partial reversal of emphysematous alterations in SIgA-deficient mice. This implies that the invasion of SIgA-deficient trachea by bacteria is a major contributor to inflammation and emphysema in pIgR-deficient (pIgR-/-) mice. In addition, leukocytes recruited to airways with acquired SIgA deficiency can produce proteases that impair the airway wall, ultimately leading to fibrosis remodeling and airflow blockage. Products of activated leukocytes, involving MMP-12 and neutrophil-derived elastase, can destroy elastic fibers and additional elements of the alveolar septa in the vicinity of these small airways, ultimately leading to lobar central emphysema.^398^ In vitro and in vivo studies indicate that indole-3-acetic acid produced by the respiratory microbiome reduces emphysema and reduces lung function through IL-22-mediated macrophage-epithelial cell interactions. Intraperitoneal injection of indole-3-acetic acid into emphysema model mice reduced the decreases in lung function, emphysema, tissue damage, collagen deposition, and levels of TNF-α, IL-1β, IL-6, and IL-17A.^223^ By comparing the differences in sputum bacteria between pneumoconiosis patients and controls, scientists found an increased percentage of *Streptococcus*, *Granuliococcus*, and *Bacillus* in pneumoconiosis patients. Meanwhile, the microbiota of coal workers’ pneumoconiosis subjects had less *Selenomonas*.^399^

Compared to numerous studies about the lung microbiome and localized lung diseases, there is a great scope for exploring the correlation between the pulmonary microbiome and systemic diseases.

## Therapeutic potential for lung disorders

**Fig. 5:**
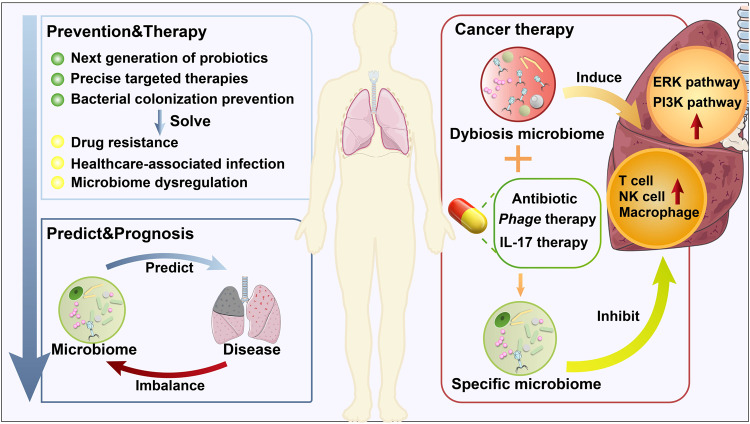
Therapeutic potential for lung disorders. The lung microbiome, as a long-standing commensal community in the lung, is involved in the development of and recovery from diseases. From disease prevention and treatment to disease prediction and prognosis and even lung cancer therapy, the lung microbiome can be further investigated as a promising target. First, clinicians can prevent health care-associated infections caused by bacteria through the discovery of next-generation probiotics. Second, by further narrowing the antimicrobial spectrum, new antimicrobial drug targets can be established to address the drug resistance of some bacteria and reduce the damage to unrelated commensal bacteria. Third, blocking bacterial colonization may reduce the ecological dysbiosis of commensal flora and prevent the onset or progression of diseases. During disease progression, the microbiome can predict disease prognosis and outcome. A dysregulated pulmonary flora often predicts worsening disease and poor outcomes, and the progression of disease further promotes dysbiosis, creating a vicious cycle that ultimately leads to an unfavorable clinical prognosis. Furthermore, the lung microbiome deeply influences the occurrence, progression, and prognosis of lung cancer. A dysregulated lung microbiome may trigger lung cancer pathogenesis through the upregulation of the ERK and PI3K pathways and upregulate Th17 cell, NK cell, and macrophage expression. The local microenvironment of tumors can be influenced by the clinical development of multiple targeted drugs. For example, these drugs can target the microbes themselves (e.g., phage) or their metabolites or target induced cytokines (e.g., IL-17) to recreate the specific microbiome and then reduce tumorigenesis or progression. (Figures are created with Servier Medical Art and exported under a paid subscription.)

The symbiotic microbiota has emerged as an important biomarker and modulator of tumorigenesis and the response to cancer therapy. It has also been widely studied as an essential tool for predicting the development and prognosis of lung diseases (Fig. 5). The microbiome-mediated pathogenesis of lung diseases has been profoundly explored from correlation to causation, especially in cancer.

### Prevention and therapy of lung diseases

Research on intestinal microbes has found that reestablishing a normal microbiome after antibiotic treatment can significantly reduce colonizing infections, especially those of antibiotic-resistant bacteria. Antibiotics have a sustained inhibitory effect, while steroids increase the relative abundance of many bacterial communities, such as members of the phylum Aspergillus (e.g., *Moraxella*, *Pasteurella*, *Pseudomonas*, and *Enterobacteriaceae*).^400^ Ongoing research explores commensal bacterial species that could be explored as the next generation of probiotics to rebuild or reinforce tolerance and prevent healthcare-associated infections resulting from highly antibiotic-resistant bacteria.^401^ By analogy, the role of the lung microbiome in disease prevention deserves attention.

Based on specific factors in the host response, such as the proliferation or reduction of specific colonies, new host-targeted approaches can be proposed for the prevention and treatment of lung diseases, such as targeting antibiotics to direct pathogenic elements of the microbial spectrum while leaving residual members of the microbial community undisturbed. Such interventions have the potential to address antimicrobial resistance, and patients may receive less antimicrobial therapy. As an example, in the treatment of hospital-acquired pneumonia, low-dose hydrocortisone reduced the risk of pneumonia in trauma patients, demonstrating the effectiveness of host-targeted therapy.^402,403^ Chronic *H. influenzae* infection contributes to Th17- and neutrophil-driven inflammation and steroid insensitivity in allergic respiratory disease. Consequently, the prevention of bacterial colonization may be a potential target for the adjuvant treatment of asthma and COPD.^216^ Influenza vaccination may also reduce secondary bacterial infections from *Streptococcus*.^404^ Since the composition, abundance, and predominant population of the lung microbiome change with the progression of the disease, in vivo, tests on the evolution of the microbiome can be performed to understand the changes in the status of the treated lungs. The most common sequencing technologies today include whole genome sequencing, NGS, and single-molecule real-time sequencing. There are differences in the diversity and abundance of microbiomes sequenced by different technological platforms, thus, the microbiome composition is influenced by the platforms and the bioinformatics pipeline.^405–407^ The viral, bacterial, and fungal compositions may be used to identify disease subtypes. Therapies targeting the microbiome may reduce the severity of the disease. For instance, chronic azithromycin therapy has been shown to reduce the frequency of COPD exacerbation, non-CF bronchiectasis, and CF.^408–410^ Probiotic administration may be an effective strategy to maintain or restore a functional microbiome, such as the use of probiotics for the prevention of allergic asthma.^411^ Several studies have examined the role of oral probiotics in the prevention of URT infections, with the majority (17 of 21) providing evidence of their beneficial effects.^412^ In vitro studies on cigarette smoke-induced diseases, such as COPD, demonstrated that the administration of *Lactobacillus rhamnosus* and *Bifidobacterium breve* eliminated the release of proinflammatory mediators from macrophages in response to cigarette smoke.^413^ Furthermore, oral administration of *Lactobacillus acidophilus* to lung cancer model mice treated with cisplatin showed a reduction in tumor volume and a higher survival rate.^414^

Scientists have conducted intensive research on the effects of gut microbial metabolites on disease and immunity. Unfortunately, the impact of lung microbiome metabolites on disease is an unexplored area. We attempted to uncover the function of metabolites of the lung microbiome, thereby providing new ideas for the diagnosis and treatment of disease. First, metabolites can be ideal biomarkers of disease progression. They confer specificity to the lung microbiome when cultured. For example, water-soluble pigments make *Pseudomonas* easy to identify. *Streptococcus* does not breakdown inulin and is not bile soluble, while *Pretus* breaks down urea and migrates for growth. Moreover, for metabolite testing to be scalable and affordable, metabolite-based diagnostics facilitate cost reduction. Second, metabolites have a bright future in the treatment of diseases. Metabolites of the gut microbiome are important coordinators of host pathophysiology based on their influence on the body’s immune, inflammatory, and other processes. SCFAs, for example, as mentioned earlier, are protectors of the organism.^415,416^ SCFAs can inhibit the inflammatory response in the lungs in a GPR41-dependent manner.^114^ SCFAs also promote thymic peripheral Treg production through different mechanisms.^417^ Butyrate suppresses proliferation as a histone deacetylase inhibitor through epigenetic regulation of gene expression, thereby enhancing protection against infection.^418–420^ In addition to SCFAs, polysaccharide A inhibits the production of proinflammatory IL-17 and promotes the expression of IL-10 by CD4 + T cells.^128,367^ However, the metabolites of the gut microbiome, which are mentioned above, have been intensively studied and systematically summarized. The metabolites of the lung microbiome, however, remain scattered. *Haemophilus* in the lung microbiome can produce polysaccharide A. Firmicutes can produce butyric acid and a small amount of propionic acid. However, scientists lack a systematic study of the metabolites of the lung microbiome. How these metabolites produced in the lungs differ from those produced in the gut and what specific effects they have on the organism deserve further investigation.

### Prognosis of lung disease

The respiratory microbiome can independently predict the prognosis of various acute and chronic lung diseases. For instance, increased pulmonary bacterial load predicts the adverse outcomes of pulmonary fibrosis^244^ and critically ill patients receiving mechanical ventilation.^393^ Reduced sputum bacterial diversity predicts mortality in COPD,^421^ and alterations in the microbial community predict deterioration of bronchiectasis^422^ and respiratory tract infections in children.^423^ In patients with bronchiectasis, differences in the respiratory microbiome were strongly correlated with the number of exacerbations that occurred in the following year.^422^ Changes in the lung microbiota also predict the response to inhaled antibiotic therapy in patients with bronchiectasis.^424^ Furthermore, pulmonary bacterial load predicted the development of chronic transplantation lung dysfunction or death within 500 days after bronchoscopy but was not specifically relevant to individual flora. This shows that the lung microbiome is a potentially modifiable but understudied risk factor for lung transplantation dysfunction.^425^

In critically ill patients, critical ecological factors affecting lung microbiota migration, elimination, and the relative colonization rates of respiratory microbiota are altered.^426^ In mechanically ventilated patients, inhalation of pharyngeal microorganisms is accelerated.^427^ Moreover, lung microbiome selectivity, such as cough, mucociliary clearance, and host immune defense mechanisms, is often impaired in critically ill patients as a result of both the disease itself and pharmacological interventions (e.g., sedation and corticosteroids).^427^ Thus, from composition to structure, the microbiome of critically ill patients differs dramatically from that of the healthy state. The most prominent bacterial taxa in the healthy respiratory microbiome (e.g., *Prevotella* and *Veillonella*) become rare in hospitalized patients. Conversely, the abundance of taxa associated with common diseases (e.g., *Enterobacter* and *Staphylococcus*) was relatively increased.^52,428^ In conclusion, ecological disturbances in critically ill patients may turn the pulmonary microbiota into a fragile ecosystem with catastrophic collapse due to the overgrowth of the dominant pathogen, which eventually results in the pathogen becoming the dominant population.

### Cancer therapy

The human microbiota plays a critical role in initiating and promoting carcinogenesis and influences the prognosis and potentially the outcome of various malignancies. Examples of related cancers include cervical cancer, gastrointestinal tumors, lung cancer, and nasopharyngeal cancer.^429^ The lung microbiome is relevant to the prognosis and treatment of lung cancer. A lack of respiratory microbiota diversity in healthy subjects is a risk factor for lung tumorigenesis.^430^ The lung adenocarcinoma-associated microbiome increases the inflammatory response by activating lung-resident T cells, while germ-free mice or mice treated with antibiotics have a lower potential of developing lung cancer due to KRAS mutation/TP53 deletion.^431^ In addition, airway microbial components may trigger lung carcinogenesis by inducing signaling pathways in oncogenes, such as *Streptococcus* and *Veillonella*-induced upregulation of the ERK and PI3K pathways.^206^ Understanding and describing the lung microbiome, particularly in patients with early-stage lung cancer, may facilitate the discovery of therapeutic biomarkers for lung cancer. To determine the feasibility of microbial DNA as a distinguishing characteristic for early-stage tumors, by using The Cancer Genome Atlas (TCGA), Poore et al. investigated the unique microbial signature present in the tissues and blood of 33 patients with primary cancer. In addition, considering the specificity of fungi in different cancers, scientists have attempted to identify features of circulating fungal DNA from 20 different fungi that may be used to distinguish pancancer from healthy individuals, demonstrating the utility of using microbiology in cancer diagnosis.^341^ Consequently, the possible use of the respiratory microbiome as a predictive biomarker is a promising area that urgently needs research.

Second, learning how the pulmonary microbiome affects the immune microenvironment in individuals with lung cancer may significantly impact the development of targeted therapies. Combining lung microbes with immunomodulatory drugs and immunotherapy may improve cancer prognosis. Therapies targeting lung cancer can aim to regulate the symbiotic lung microbiota to prompt a more tumor-suppressive environment. Inducing the microbiota to target the production of bacterial enzymes or byproducts is essential for tumorigenesis and the tumor immune response. These factors regulate the host biological effects produced by the pulmonary microbiome or the mechanisms that target the migration of bacteria into the lung. For example, intraosseous injection of vancomycin into mice resulted in altered lung microbiota,^432^, and intranasal infusion of *Lactobacillus* can activate respiratory immunity and improve resistance to viral infections.^179^ Analogously, the reduction in flora induced by nebulized antibiotic exposure decreased the implantation of tumors in mouse lungs and dramatically reduced lung metastasis.^178^ Furthermore, phages may be clinically studied against specific lung microbiota to transform lung cancer patients’ tumors and immune microenvironments into a more tumor-resistant environment.^433^ In addition, by understanding the composition of pulmonary commensal flora in lung cancer at the species or genus level, metabolic pathways present in the altered commensal community were examined for the development of targeted therapeutic agents against the metabolites. This type of altered metabolite, such as bacterial enzymes, in turn affects the local tumor microenvironment. Clinical research has shown that metabolites or peptides produced by the gut microbiome can act as ligands, which interact with host receptors and trigger downstream signaling. However, studies targeting the lung microbiome have not been completed.^434^ Owing to Th17 induction by the lung microbiome, combined with the fact that lung cancer is distinguished by an immune microenvironment rich in Th17 cell responses and IL-17 and other cytokine expression, we speculate that targeting IL-17 in combination with immunotherapy may improve the therapeutic responses of lung cancer patients. As an example, when treated with neutralizing antibodies, mice showed a significant reduction in IL-1β, tumor growth, and neutrophil infiltration.^200^ Up-to-date research has paid attention to the relationship between the lung microbiome and immune checkpoint inhibitor therapy. Increasing microbiome leads to poor therapeutic effect of anti-PD-1 therapy, as proved by certain research. However, the effect of immune checkpoint inhibitor therapy on the microbiome has not been systematically concluded.

## Conclusions and future perspectives

In this review, we retrospectively reviewed the history of research from the discovery of the lung microbiome and its composition, role, and connection with respiratory diseases. Most importantly, comprehensive research was undertaken to survey the effect of the microbiome on lung cancer. The theory that the lung was once considered a completely sterile site was gradually corrected with the development of sequencing technology. The lung microbiome was low in number and abundance compared to microbial communities in other sites, such as the intestine and the oral cavity. The main lung microbiome can be divided into the bacterial group, the mycobiome, and the virome. Most of the microorganisms in the lungs originate from the URT, such as *Ralstonia*, and are similar in composition to the oropharyngeal microbiome. Nevertheless, lung microbes have specific flora, such as *Haemophilus*, which the oral cavity does not. The lung microbiome of a healthy organism plays an important role in the maintenance of lung homeostasis by regulating the lung environment and modulating the immune response. The lung microbiome is not a permanent, stable collection of colonies but is fluid. This means that the lung microbiome is closely linked to the oral and intestinal microbiomes and they influence each other. Alterations or shifts in the oropharyngeal microbiome will affect the composition of the lung microbiome, while the gut-lung axis links the intestinal and lung microbiomes, affecting the whole body in one way or another. The gut-lung axis reveals the metabolic and immunologic relationship between these two organs. The gut microbiome regulates the immune response of the lung microbiome through the production of metabolites, such as SCFAs, while the lung microbiome can also influence the development of intestinal diseases through alterations in the gut microbial community. However, the exploration of the gut-lung axis is far from over. The genetic role of microorganisms and their metabolites in the lung has not yet been investigated. After decades of research, researchers have discovered a connection between the lung microbiome and a variety of lung diseases. These include asthma, COPD, CF, and the recent occurrence of COVID-19. The microbiome of the diseased lung is significantly different from that of the healthy state, and the number and abundance of the dominant genera, flora, and bacteria vary according to the disease. In turn, disorders of the lung microbiome contribute to the onset and exacerbation of diseases. The association between lung cancer and the microbiome has recently become a new trend. As a malignant lung disease, lung cancer is a major challenge for treatment. Researchers have found that the lung microbiome profoundly impacts the tumorigenesis, progression, and prognosis of lung cancer. Cellular DNA damage due to dysbiosis of the lung microbiome and chronic inflammation due to metabolites are triggers for lung cancer development. Moreover, increased microbiome abundance during cancer leads to decreased immunotherapeutic efficacy, and a high bacterial load leads to poor cancer prognosis. It is evident that the microbiome is involved in the whole process of lung cancer, and its changes and activities control the course of cancer. The microbial composition of lung cancer is different from that of healthy organisms, in which the metabolites of the microbiome, such as bacterial toxins, may be markers of lung cancer and help in clinical diagnosis and treatment.^338^

Looking ahead, we think about many more possibilities in the field of the lung microbiome. First, lung microbes and their metabolites could become diagnostic markers of disease progression. Different diseases, such as asthma and COPD, have different variations in microbiomes. The quantitative changes in the specific microbiome can assist in the rapid clinical diagnosis of disease and timely follow-up of treatment progress. For example, in a prospective cohort of critically ill patients receiving mechanical ventilation, increased bacterial load predicted a decrease in ventilator-free days.^435^ The metabolites are inexpensive and suitable for the diagnosis of lung diseases. In addition, metabolites can be administered with easily controlled pharmacokinetics, thereby reducing the risk of immune reactions associated with drugs. Second, in the emerging field of lung transplantation, the lung microbiome can predict chronic rejection or death.^436,437^ Bacterial DNA load is an independent predictor of poor prognosis in critically ill patients. Poor ICU prognosis can be forecast by the composition of the pulmonary microbiota of critically ill patients.^393^ Third, in the study of lung cancer, the lung microbiome can provide targets for tracking dynamic changes in cancer by its mobile, transient colonization characteristics. Furthermore, unlike other major sources of cancer heterogeneity, the lung microbiome can be easily modified by clinical intervention. The respiratory microbiome is a promising therapeutic target as a potential “treatable trait” for subphenotypic patients and for utilizing precision medicine.^438^ The addition of microbiome-specific therapies to the treatment of cancer can improve patient survival and prognosis.

Nevertheless, we still need to address the shortcomings of this research area. Most researchers report that sampling of the lung microbiome is flawed, i.e., oral and respiratory microbial contamination cannot be excluded.^52,69,439^ This limitation has resulted in the composition and function of the lung microbiome remaining incompletely characterized. Although current sequencing technologies, such as NGS, can identify most of the microbial communities present in the lung, there are still deficiencies. The detection of fungi and viruses is much more challenging than that of bacteria, so we lack a thorough study of these two members. There is still an absence of research on the correlation and interaction mechanisms between the lung microbiome and lung metastasis of other organ-derived tumors, such as breast cancer. Furthermore, our knowledge of the epigenetic-metabolic-lung microbiome relationship is still lacking. The effects of pulmonary microbial metabolites on the lungs and the whole body have not been systematically investigated. In the future, we should adopt more precise cutting-edge techniques to deeply explore the composition and role of the mycobiome and virome. More importantly, integrated biological approaches are required to explore the mechanisms of interaction between bacteria, fungi, and viruses. Determining the connection between lung metabolites and the immune system and the relationship between genes and lung microorganisms still require more research. Finally, we should expand the field of lung microbiology and diseases research to explore the connection between the lung microbiome and cancer development and metastasis at other sites and to evaluate the value of the lung microbiome more comprehensively.
